# Chemical Profiles of the Oxides on Tantalum in State of the Art Superconducting Circuits

**DOI:** 10.1002/advs.202300921

**Published:** 2023-05-11

**Authors:** Russell A. McLellan, Aveek Dutta, Chenyu Zhou, Yichen Jia, Conan Weiland, Xin Gui, Alexander P. M. Place, Kevin D. Crowley, Xuan Hoang Le, Trisha Madhavan, Youqi Gang, Lukas Baker, Ashley R. Head, Iradwikanari Waluyo, Ruoshui Li, Kim Kisslinger, Adrian Hunt, Ignace Jarrige, Stephen A. Lyon, Andi M. Barbour, Robert J. Cava, Andrew A. Houck, Steven L. Hulbert, Mingzhao Liu, Andrew L. Walter, Nathalie P. de Leon

**Affiliations:** ^1^ Department of Electrical and Computer Engineering Princeton University Princeton NJ 08544 USA; ^2^ Center for Functional Nanomaterials Brookhaven National Laboratory Bldg. 735, P.O. Box 5000 Upton NY 11973‐5000 USA; ^3^ Materials Measurement Science Division, Material Measurement Laboratory National Institute of Standards and Technology Gaithersburg MD 20899 USA; ^4^ Department of Chemistry Princeton University Princeton NJ 08544 USA; ^5^ Department of Physics Princeton University Princeton NJ 08544 USA; ^6^ National Synchrotron Light Source II Brookhaven National Laboratory Bldg 740 Upton NY 11973‐5000 USA

**Keywords:** dielectric loss, oxide, qubits, superconducting thin films, tantalum, X‐ray photoelectron spectroscopy

## Abstract

Over the past decades, superconducting qubits have emerged as one of the leading hardware platforms for realizing a quantum processor. Consequently, researchers have made significant effort to understand the loss channels that limit the coherence times of superconducting qubits. A major source of loss has been attributed to two level systems that are present at the material interfaces. It is recently shown that replacing the metal in the capacitor of a transmon with tantalum yields record relaxation and coherence times for superconducting qubits, motivating a detailed study of the tantalum surface. In this work, the chemical profile of the surface of tantalum films grown on c‐plane sapphire using variable energy X‐ray photoelectron spectroscopy (VEXPS) is studied. The different oxidation states of tantalum that are present in the native oxide resulting from exposure to air are identified, and their distribution through the depth of the film is measured. Furthermore, it is shown how the volume and depth distribution of these tantalum oxidation states can be altered by various chemical treatments. Correlating these measurements with detailed measurements of quantum devices may elucidate the underlying microscopic sources of loss.

## Graphical abstract

Tantalum superconducting qubits are a leading candidate for large‐scale quantum computing. These devices are currently limited by two‐level‐systems at material surfaces and interfaces whose microscopic origin is unknown. This study uses variable energy X‐ray photoelectron spectroscopy to determine the chemical profile of the tantalum oxide along its depth, which allows comparison with device measurements to elucidate microscopic loss mechanisms.

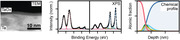

## 1 Introduction

Superconducting qubits are the basis of many efforts to build large scale quantum computers, and have enabled key demonstrations of quantum algorithms,^[^ ^1^ ^]^ quantum error correction,^[^ ^2^, ^3^, ^4^, ^5^, ^6^ ^]^ quantum many body physics,^[^ ^7^, ^8^, ^9^, ^10^ ^]^ and quantum advantage in performing specific tasks.^[^ ^11^ ^]^ Despite this activity, little progress has been made in identifying and addressing the microscopic source of loss and noise in the constituent materials. The lifetimes of current 2D transmons are believed to be limited by microwave dielectric losses.^[^ ^12^, ^13^ ^]^ Recent work has measured the dielectric loss tangent of high‐purity bulk sapphire as 15(5) × 10^−9^ at qubit operating conditions.^[^ ^14^ ^]^ This loss tangent would result in a qubit lifetime of several milliseconds if it were the only source of dielectric loss, suggesting that losses are dominated by uncontrolled defects at surfaces and interfaces or by material contaminants.^[^ ^15^ ^]^

We have recently demonstrated that tantalum (Ta) based planar transmon qubits can exhibit record lifetimes over 0.3 ms and quality factors (Q) over seven million,^[^ ^12^ ^]^ exceeding the prior established record of niobium (Nb) and aluminum (Al) based planar transmon qubits by a factor of three. Other groups have recently realized Ta qubits with Q over 10 million and resonators with low power Q over four million.^[^ ^13^, ^16^ ^]^ We hypothesize that one key advantage of Ta is its robust, stochiometric oxide, which is resistant to a wide range of aggressive chemical processes.^[^ ^17^, ^18^ ^]^ These observations motivate a careful study of the oxide species at the surface of Ta, as the amorphous oxide layer at the surface is a plausible source of dielectric loss arising from two‐level systems.^[^ ^19^, ^20^ ^]^ We have recently reported measurements on superconducting resonators showing that two‐level systems at material interfaces are the dominant source of dielectric loss for tantalum devices, and that some of these two‐level system defects reside in the surface oxides of tantalum.^[^ ^21^ ^]^ In that work, we estimated that the oxide layer is responsible for around half of the surface‐related losses in state‐of‐the‐art devices by using detailed comparisons between devices treated with different acids. In this work, we investigate the nature of the tantalum oxide resulting from these chemical treatments.

In this study, we use variable energy X‐ray photoelectron spectroscopy (VEXPS) to characterize the surface of Ta thin films employed in state‐of‐the‐art superconducting circuits, with the aim of identifying possible microscopic sources of loss and noise. We measure and analyze Ta4f ionizations with different peaks across a range of incident X‐ray energies to vary the sampling depth and use the combined dataset to build a depth profile of the different oxide stoichiometries. We show that the Ta surface is dominated by the fully oxidized Ta_2_O_5_, but that between the Ta_2_O_5_ surface layer and the bulk metal, suboxide species containing Ta^3 +^ and Ta^1 +^ are present. Compared to similar measurements of the oxides of Nb,^[^ ^22^ ^]^ the Ta oxide layer is thinner and the interfaces are more abrupt. We apply our measurement and analysis technique to films treated with different acid processes and show that it is possible to controllably grow and shrink the Ta oxide layers.

## 2 Experimental Section

**Figure 1:**
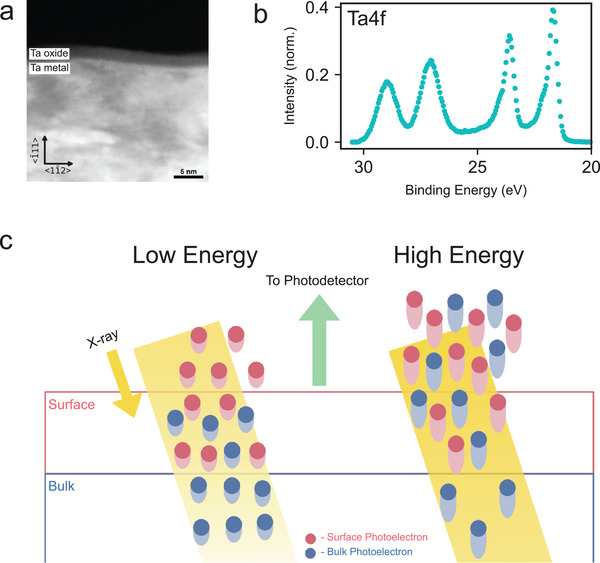
a) High angle annular dark field scanning transmission electron microscope image of the cross‐section of a tantalum film on sapphire. The tantalum film has a BCC crystal structure and was grown in the (111) orientation on a c‐plane sapphire substrate. An amorphous oxide layer can be seen on top of the tantalum at the tantalum air interface. b) Experimental results of the tantalum binding energy spectrum obtained from X‐ray photoelectron spectroscopy (XPS) performed using 760 eV incident photon energy. Each oxidation state of tantalum contributes a pair of peaks to the spectrum due to spin‐orbit splitting. At the highest binding energy (26–30 eV), there is a pair of peaks corresponding to the Ta^5 +^ state. At the lowest binding energy, we see a pair of sharp asymmetric peaks corresponding to metallic tantalum (21–25 eV). c) Schematic explaining the physics behind variable energy X‐ray photoelectron spectroscopy (VEXPS). The red and blue dots correspond to photoelectrons excited from a surface oxidation state and bulk oxidation state of the tantalum films respectively. When low energy X‐rays are incident on the film surface, photoelectrons are excited with low kinetic energy (depicted by a small tail on the dots). These low energy photoelectrons have a shorter mean free path so that only those emitted from the surface species (colored red) will exit the material and impinge on the detector. When high energy X‐rays are incident on the film surface, photoelectrons with high kinetic energy are excited (depicted by a longer tail on the dots). These higher energy photoelectrons have comparatively longer mean free paths so that electrons from the bulk of the film will exit the material alongside electrons from the surface. In our experiment, the angle between the surface and the incident X‐rays varies between 6° and 10°; the X‐rays in this image are shown at a steeper angle for legibility.

All experiments used 200 nm thick α‐Ta films grown on 500 µm thick c‐plane sapphire substrates by DC magnetron sputtering.^[^ ^12^, ^21^ ^]^ A typical tantalum film is covered by a thin layer of amorphous oxide, as shown by its cross‐sectional scanning transmission electron microscope (STEM) image (**Figure** **1**a). All samples studied throughout the main text of this paper were from the same deposition, which has a body‐centered cubic (bcc) crystal structure (α phase) with a (111) out‐of‐plane orientation. All samples were cleaned in a 2:1 H_2_SO_4_:H_2_O_2_ piranha bath for 20 min (Section S1, Supporting Information).

X‐ray photoelectron spectroscopy (XPS) was used to study the oxide at the surface of Ta (Figure 1b). In the 20 to 30 eV binding energy range, two prominent pairs of peaks associated with Ta4f core electron ionization were observed. Each oxidation state of Ta appears as a doublet attributed to Ta4f_7/2_ and Ta4f_5/2_ because of significant spin‐orbit coupling.^[^ ^23^ ^]^ The pair of peaks at 22 and 24 eV have been previously assigned to metallic Ta^0^, while the pair at 27 and 29 eV were assigned to Ta^5 +^, here in the form of Ta_2_O_5_. The full width half maximum linewidth of the metallic peaks (approximately 0.5 eV) was narrower than those of the oxide (approximately 1.0 eV), likely arising from a higher degree of disorder in the oxide, which was amorphous (Figure 1a). Shoulder peaks on the higher binding energy side of the Ta^0^ peaks were also observed. In addition to these resolvable peaks, there was a broad baseline in the binding energy range between the Ta^0^ and Ta^5 +^ peaks, arising from intermediate oxidation states of Ta. In general, a single XPS scan was insufficient to constrain the number of peaks and their associated linewidths, and it does not provide information on how the different oxidation states of tantalum were distributed through the depth of the film.

In order to disentangle the different oxidation states and study their spatial distribution, VEXPS using 17 different incident photon energies in the range from 630 to 6000 eV was performed. At lower photon energies, photoelectrons have lower kinetic energy and shorter inelastic mean free paths (IMFP); therefore, lower photon energies were more surface sensitive.^[^ ^23^ ^]^ The kinetic energy and the IMFP increase with increasing photon energy; thus, the XPS measurements become more bulk sensitive (Figure 1c). The surface sensitivity of the low incident energy scans by using grazing incidence was enhanced, which further boosts the depth sampling contrast between photon energies due to the attenuation of X‐ray photons through the material. By studying the relative fractions of photoelectrons collected from different oxidation states at different incident photon energies, the depth distribution of the various tantalum oxidation states was inferred.

## 3 Results and Discussion

To quantify the relative abundance of each oxide species, we fit the full dataset of VEXPS spectra with different energies simultaneously. Fitting all spectra at once constrains the relative peak positions, relative peak intensities, peak widths, and skewnesses, which reduces the number of free fit parameters and increases our confidence in the fitted photoelectron intensities. In addition to the Ta^5 +^ and Ta^0^ doublets, another three doublets of lower intensity are required to satisfactorily fit the spectra for all photon energies. These include one doublet at approximately 0.4 eV higher binding energy than the Ta^0^ doublet, which is most prominent at intermediate energies, and two sets of doublets that cannot be individually resolved, located between 21 and 22 eV (Ta4f_7/2_) and between 24 and 26 eV (Ta4f_5/2_).

**Figure 2:**
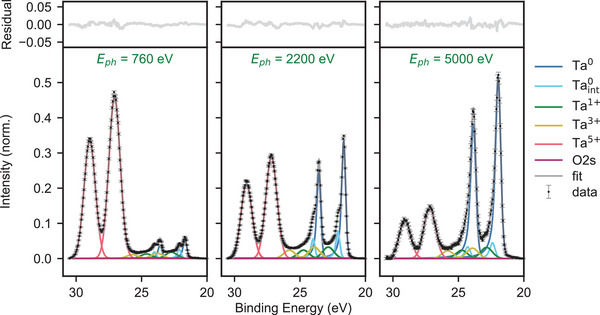
Shirley background corrected XPS spectra of Ta4f binding energy obtained at three different incident photon energies. Left panel: with 760 eV X‐ray photons, the Ta^5 +^ peaks dominate over the Ta^0^ peaks. Middle panel: at 2200 eV photon energy, there is almost equal contribution of photoelectrons at Ta^0^ and Ta^5 +^. Right panel: At 5000 eV photon energy, the dominant photoelectron contribution is coming from Ta^0^. In all three plots there is non‐zero intensity between the Ta^5 +^ and metallic tantalum peaks, indicating minority tantalum oxidation states. The complete set of data and fits corresponding to all 17 incident X‐ray energies is shown in Section S3.3 (Supporting Information). The data are fit with Gaussian profiles for the Ta^5 +^, Ta^3 +^, and Ta^1 +^ species, and skewed Voigt profiles for the Ta^0^ and Ta${}_{\text{int}}^{0}$. Included in the fit is also a Gaussian profile corresponding to the O2s peak; the amplitude of this peak is fixed to 5% of the measured O1s peak intensity.

The Ta^0^ peaks and their shoulder peaks offset by approximately 0.4 eV have similar linewidths, indicating that they both arise from metallic states, so we fit them both with skewed Voigt profiles.^[^ ^24^ ^]^ All other peaks that exhibit much broader linewidths are fit with Gaussian profiles.^[^ ^24^ ^]^ A Shirley background correction is applied to all data prior to XPS peak fitting,^[^ ^25^ ^]^ and the data are normalized so that the total intensity under the curve is unity. Lastly, we account for the O2s photoelectron emission that overlaps with Ta4f peaks^[^ ^23^ ^]^ by measuring the O1s peak. We use the ratio of the photo‐ionization cross‐sections for the O1s and O2s photoelectrons^[^ ^26^ ^]^ to estimate the photoelectron intensity of the O2s peak, neglecting the impact of the kinetic energy difference between O1s and O2s photoelectrons. We find the contribution from the O2s peak to be only a few percent of the total photoelectron intensity for all energies. The background corrected spectra and the results of our peak fitting algorithm are shown in **Figure** **2** for three different photon energies. The Ta^0^ peak intensities increase with increasing photon energy, while the Ta^5 +^ peak intensities decrease, as expected for an oxide layer at the surface. More information on the fitting procedure, as well as the data and fits for all spectra, can be found in Section S3 (Supporting Information).

We identify the doublet shifted by 0.4 eV from the Ta^0^ doublet as the layer of tantalum metal closest to the metal‐oxide interface, as described in Ref.[27] The differing coordination number of tantalum atoms in this layer results in a higher binding energy. We denote this interface species as Ta${}_{\text{int}}^{0}$. We identify the two remaining doublets between 22 and 26 eV as Ta^1 +^ and Ta^3 +^ species based on their locations relative to the Ta^0^ and Ta^5 +^ peaks.^[^ ^27^ ^]^ These additional oxidation states could arise from suboxides of tantalum or other materials, such as tantalum nitride or tantalum carbide. In a wide XPS survey scan we detect no significant atomic species other than tantalum, oxygen, and carbon, thus excluding the presence of nitrides. In a different sample from the same film, we sputtered the top layer with an in‐situ argon ion mill, and found that when the C1s peak was removed, the O1s spectrum was largely unchanged, and the Ta^1 +^ and Ta^3 +^ peaks remained (Section S5, Supporting Information). Thus the carbon is present only as adventitious carbon on the top surface of the film, and the Ta^1 +^ and Ta^3 +^ species arise from suboxides. We therefore assign the Ta^1 +^ and Ta^3 +^ species as amorphous Ta_2_O and Ta_2_O_3_, respectively.

We obtain a depth dependent chemical profile of the oxide by modeling the dependence of the photoelectron intensity fraction of each species on the incident photon energy. The sample is modeled as a multi‐component thin film with five distinct species. Of these five species, the bottom species, Ta^0^, is assumed to have thickness beyond the sampling depth of our measurements, which we model as being infinite. We assume that the sample composition varies only along the depth *x*, and that it is homogeneous across the plane. The five species are spatially mixed through the depth, described by a set of depth‐dependent volume fractions, {*F*_*n*_(*x*)}, with *n* indexing the 5 different species. The volume fractions are constrained by the sum rule of $\sum_{n=1}^{5}F_{n}\left(x\right)=1$ and a limiting case for the tantalum metal substrate, $\lim_{x\to \infty }F_{{\text{Ta}}^{0}}\left(x\right)=1$.

At each photon energy *E*_*ph*_, the intensity fraction, *W*_*n*_, of photoelectrons from species *n* is obtained by normalizing its photoelectron flux *A*_*n*_(*E*_*ph*_) against the total flux, that is,

(1) $$W_{n}\left(E_{ph}\right)=\frac{A_{n}\left(E_{ph}\right)}{\sum_{m=1}^{5}A_{m}\left(E_{ph}\right)}$$

The photoelectron flux *A*_*n*_ can be computed for each given set of volume fractions {*F*_*n*_(*x*)}, by considering the attenuation of both the X‐rays and the photoelectrons along their respective travel paths:

(2) $$\begin{array}{ccc} A_{n}\left(E_{ph}\right) & = & \gamma \eta \rho_{n}\int_{0}^{\infty }F_{n}\left(x\right)\exp[-\int_{0}^{x}\frac{\mu (E_{ph})}{\rho_{0}}\frac{\rho (s)}{\sin\Theta_{ph}}ds\\ & & \;\;\;\;\;-\;\frac{x}{\lambda_{el}\left(E_{el}\right)\sin\Theta_{el}}]dx \end{array}$$

where γ is the photoelectron collection efficiency; η is the photoelectron yield; ρ_*n*_ is the density of species *n*; $\rho \left(s\right)=\sum_{n}F_{n}\left(s\right)\rho_{n}$ is the total density at depth *s*; μ/ρ_0_ is the X‐ray mass attenuation coefficient; Θ_*ph*_ is the angle between the X‐ray beam and the sample surface; Θ_*el*_ is the angle between the electron detector and the sample surface; λ_*el*_ is the effective electron attenuation length; and *E*_*el*_ = *E*_*ph*_ − *E*_*b*_ is the kinetic energy of the photoelectron, where *E*_*b*_ is the binding energy of the core level. Derivation of Equation 2 is detailed in Section S4.2 (Supporting Information). Further discussion can be found in Ref.[28]

Several parameters in Equation 2 are needed to properly evaluate the intensity fraction *W*_*n*_ in Equation 1. Our experimental configuration has Θ_*el*_ = 80° and Θ_*ph*_ = 6° (=10°) for *E*_*ph*_ < 2000 eV (⩾2000 eV). The values of photoelectron yield η and photoelectron collection efficiency γ are assumed to be independent of species, so that they cancel in computing *W*_*n*_. The values for μ(*E*_*ph*_)/ρ_0_ are obtained from those tabulated in Ref.[29] The effective electron attenuation length λ_*el*_ is assumed to be independent of the tantalum species and computed using the empirical relation^[^ ^30^ ^]^

(3) $$\lambda_{el}\left(E_{el}\right)=\lambda_{im}\left(E_{el}\right)\left[1-0.836\omega \left(E_{el}\right)\right]$$

as a function of electron kinetic energy *E*_*el*_, where λ_*im*_ is the inelastic mean free path of electrons in tantalum and ω is the single‐scattering albedo of tantalum, both tabulated versus the electron kinetic energy.^[^ ^30^, ^31^ ^]^ To further simplify the computation, it is noted that *E*_*b*_ in Equation 2 is well approximated by the average binding energy of all tantalum species (24 eV), because *E*_*b*_ does not vary appreciably and *E*_*ph*_ ≫ *E*_*b*_. As such, we use *E*_*el*_ ≡ *E*_*ph*_ − 24 eV (neglecting work function differences).

In principle, the volume fractions {*F*_*n*_(*x*)} can be obtained by fitting *W*_*n*_(*E*_*ph*_) specified by Equation 1 and Equation 2 to the experimental photoelectron intensity fractions. In practice, a parameterization of {*F*_*n*_(*x*)} is needed to limit the number of independent fitting parameters and to avoid overfitting. {*F*_*n*_(*x*)} are parameterized using a basis set of smooth, analytic functions that are normalized by themselves, that is, $\sum_{n=1}^{5}F_{n}\left(x\right)=1$ for all depth *x* (Section S4.3, Supporting Information).

**Figure 3:**
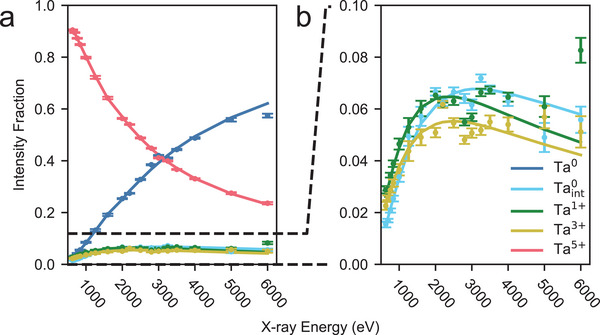
a) Relative photoelectron fraction for the different tantalum oxidation states as a function of incident X‐ray photon energy. The dots are experimental data extracted from fitting Ta4f spectra at different incident X‐ray energies. Solid lines represent an iterative fit to the data using Equations 1 and 2, corresponding to the expected intensity fractions from the depth profile shown in Figure 4a. b) Close up view of relative photoelectron fraction contribution of Ta^3 +^, Ta^1 +^, and Ta${}_{\text{int}}^{0}$. The rise, plateau, and fall of the photoelectron intensity fractions with X‐ray energy indicate that these three species are localized at a buried interface.

**Figure 4:**
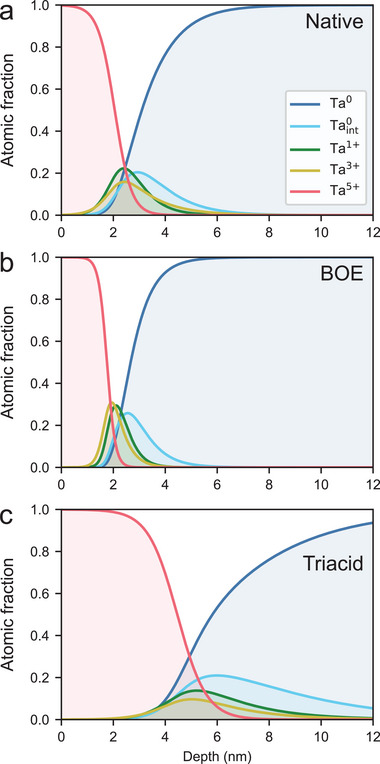
Extracted chemical profiles for three different samples: a) native b) BOE treated and c) triacid treated. Fitted photoelectron intensity fractions are shown in Section S4.6 (Supporting Information).

For a film that has only undergone piranha cleaning (“native”) film, the photoelectron intensity fraction of Ta^0^ increases monotonically and that of Ta^5 +^ decreases monotonically with photon energy (**Figure** **3**a). The photoelectron intensity fractions of Ta^1 +^, Ta^3 +^, and Ta${}_{\text{int}}^{0}$ initially increase with photon energy, but then peak and slowly decrease (Figure 3b). These observations indicate that the Ta^0^ species is located in the bulk, the Ta^5 +^ species is located at the surface, and the other species are located at an intermediate depth. The resulting depth profile shows a surface layer of Ta^5 +^ which extends approximately 2 nm into the depth, an approximately 1 nm to 2 nm thick region with overlapping Ta^3 +^, Ta^1 +^, and Ta${}_{\text{int}}^{0}$ profiles, and a bulk layer of Ta^0^ (**Figure** **4**a).

Quantitative depth profiling allows us to make detailed comparisons between different films. In addition to measuring the native tantalum oxide after our sputter deposition process, we also measure the tantalum oxide after two different surface treatments. The first is immersing the film for 20 min in 10:1 buffered oxide etch (BOE) to etch the oxide, and the second is refluxing the film in 1:1:1 nitric, perchloric, and sulfuric acids for 2 h (“triacid”) to grow the oxide (Section S2, Supporting Information). The results of the depth profile analysis for each case are shown in Figure 4b,c with the fitted photoelectron intensities shown in Section S4.6 (Supporting Information). We also extract an effective thickness of each tantalum oxide species and the tantalum interface species by integrating the area under each curve. The effective thicknesses for the native, BOE treated, and triacid treated films are tabulated in **Table** **1**.

**Table 1:** Effective thickness of different tantalum oxidation states as obtained from depth profile fitting for different tantalum films. All data in nm. Uncertainties are ±1σ confidence intervals reflecting uncertainty in our fit

Film	Ta^5 +^	Ta^3 +^	Ta^1 +^	Ta${}_{\text{int}}^{0}$
Native	2.257 ± 0.023	0.370 ± 0.016	0.370 ± 0.017	0.368 ± 0.019
Ta treated in 10:1 BOE	1.853 ± 0.028	0.296 ± 0.022	0.302 ± 0.023	0.400 ± 0.021
Ta treated in triacid	4.826 ± 0.036	0.379 ± 0.016	0.545 ± 0.020	1.198 ± 0.027

The BOE treated film exhibits statistically significantly smaller effective thicknesses than the native film for the Ta^5 +^, Ta^3 +^, and Ta^1 +^ species (approximately 20% lower in all cases), while the Ta${}_{\text{int}}^{0}$ layer effective thickness does not show a significant difference (Table 1 and Figure 4). The triacid treated film shows significantly larger thicknesses for all four species compared to either the native or BOE treated film. Furthermore, the BOE treated film has a narrower distribution of the Ta^1 +^, Ta^3 +^, and Ta${}_{\text{int}}^{0}$ species compared to the native film while the triacid film has significantly broader distributions of those three species.

Based on our measured etch rate, the BOE treatment does not etch away the entire Ta^5 +^ layer (Section S6, Supporting Information), and the mechanism for BOE interaction with the buried interface is unclear. Using atomic force microscopy (AFM) on the film profiled in Figure 4a, we observe a root mean square surface roughness of 0.568 nm with a minimum observed depth of 1.8 nm across a 500 nm square area (Section S7, Supporting Information). These two values are a significant fraction of the 2.257 ± 0.023 nm thick Ta^5 +^ layer, we found for our native oxide film. We have also observed small pinholes of similar depth variation in samples with smoother morphology, which exhibit the same chemical profile in VEXPS (Section S7, Supporting Information). We hypothesize that surface roughness and pinholes allow access to the buried interface. The X‐ray spot size in our VEXPS experiments was an ellipse with major and minor diameters approximately 300 and 50 µm, which is much larger than the surface roughness features we observe. Therefore the VEXPS measurements reflect an average over many of the surface roughness features.

We can use VEXPS data to elucidate potential sources of microwave loss. In Ref.,[21] using data from native, BOE treated, and triacid treated samples, we estimated that if the microwave dielectric loss tangent of the tantalum oxide scales with the thickness of the oxide layer, then contributions to the microwave dielectric loss tangents from the tantalum oxide and adventitious carbon species are comparable. However, the chemical profiles of the native, BOE treated, and triacid treated samples show that the thicknesses and distributions of each of the Ta^5 +^, Ta^3 +^, Ta^1 +^, and Ta${}_{\mathrm{int}}^{0}$ species vary with surface treatments. Therefore changes in the dielectric loss tangent could arise from changes to any combination of the interface species. For example, based on these detailed chemical profiles, an alternative plausible hypothesis for the observations in Ref.[21] would be that the dielectric loss arises entirely from the Ta^3 +^ layer rather than separate contributions of the oxide and adventitious hydrocarbons. Independently varying the thickness and distribution of the Ta^5 +^, Ta^3 +^, Ta^1 +^, and Ta${}_{\mathrm{int}}^{0}$ over many more surface compositions than those measured in Ref.[21] could be used to determine a precise, atomistic model for dielectric loss.

In addition to comparing depth profiles across differently treated tantalum films, we can compare the depth profile of the native tantalum film (Figure 4a) to a similar depth profile from a niobium surface presented in Ref.,[22] in which depth profiles of various niobium films were measured and compared to coherence times of qubits measured on each film. The total oxide layer thickness is appreciably smaller in tantalum versus niobium, and the tantalum suboxide species are more confined to the oxide‐metal interface. These differences suggest that the comparatively thinner oxide and more confined suboxide layer of tantalum may be one reason why qubits made out of tantalum films show longer coherence times than those made out of niobium films. This observation is consistent with recent measurements of niobium resonators with thinner oxide and correspondingly higher quality factors.^[^ ^32^ ^]^

## 4 Conclusion

Variable energy X‐ray photoelectron spectroscopy and chemical profiling reveals that in addition to the dominant Ta^5 +^ oxide and previously known Ta${}_{\text{int}}^{0}$ species, there also exist two tantalum suboxide species that we have identified as Ta^1 +^ and Ta^3 +^. These two suboxides are localized in depth at the interface between the Ta^5 +^ layer and the bulk Ta^0^. We observe that the tantalum metal‐air interface contains a smaller fraction of minority species and has a thinner majority oxide species than a corresponding Nb interface,^[^ ^22^ ^]^ which could explain the improved performance of tantalum‐based superconducting devices. Our measurements on BOE treated and triacid treated films show that the tantalum oxide is surprisingly robust, but can be altered slightly. Correlating these measurements with systematic measurements of quantum devices based on Ta films indicate that the Ta^3 +^ species may be the source of the dielectric loss currently limited state‐of‐the‐art devices. More surface treatments which vary the thickness and distribution of the species on the Ta surface could investigate this hypothesis and guide future work on device optimization. More broadly, our method of data collection and analysis can provide a foundation for future studies of the interface layers of tantalum thin films or thin films of other metals to understand the structure of those interfaces and effects of surface treatments.

## Conflict of Interest

The authors declare no conflict of interest.

## Supporting information

Supporting Information

## Data Availability

The data that support the findings of this study are openly available in Harvard Dataverse at https://doi.org/10.7910/DVN/N2WAZN, reference number 1.

## References

[1] A. Kandala , A. Mezzacapo , K. Temme , M. Takita , M. Brink , J. M. Chow , J. M. Gambetta , Nature 2017, 549, 242. 28905916 10.1038/nature23879

[2] A. D. Córcoles , E. Magesan , S. J. Srinivasan, A. W. Cross , M. Steffen , J. M. Gambetta , J. M. Chow , Nat. Commun. 2015, 6, 6979. 25923200 10.1038/ncomms7979 PMC4421819

[3] M. Gong , X. Yuan , S. Wang , Y. Wu , Y. Zhao , C. Zha , S. Li , Z. Zhang , Q. Zhao , Y. Liu , F. Liang , J. Lin , Y. Xu , H. Deng , H. Rong , H. Lu , S. C. Benjamin , C.‐Z. Peng , X. Ma , Y.‐A. Chen , X. Zhu , J.‐W. Pan , Natl. Sci. Rev. 2022, 9, nwab011. 35070323 10.1093/nsr/nwab011 PMC8776549

[4] J. Kelly , R. Barends , A. G. Fowler , A. Megrant , E. Jeffrey , T. C. White , D. Sank , J. Y. Mutus , B. Campbell , Y. Chen , Z. Chen , B. Chiaro , A. Dunsworth , I.‐C. Hoi , C. Neill , P. J. J. O'Malley , C. Quintana , P. Roushan , A. Vainsencher , J. Wenner , A. N. Cleland , J. M. Martinis , Nature 2015, 519, 66. 25739628 10.1038/nature14270

[5] M. D. Reed , L. DiCarlo , S. E. Nigg , L. Sun , L. Frunzio , S. M. Girvin , R. J. Schoelkopf , Nature 2012, 482, 382. 22297844 10.1038/nature10786

[6] V. V. Sivak , A. Eickbusch , B. Royer , S. Singh , I. Tsioutsios , S. Ganjam , A. Miano , B. L. Brock , A. Z. Ding , L. Frunzio , S. M. Girvin , R. J. Schoelkopf , M. H. Devoret , Nature 2023, 616, 50. 36949196 10.1038/s41586-023-05782-6

[7] X. Mi , M. Ippoliti , C. Quintana , A. Greene , Z. Chen , J. Gross , F. Arute , K. Arya , J. Atalaya , R. Babbush , J. C. Bardin , J. Basso , A. Bengtsson , A. Bilmes , A. Bourassa , L. Brill , M. Broughton , B. B. Buckley , D. A. Buell , B. Burkett , N. Bushnell , B. Chiaro , R. Collins , W. Courtney , D. Debroy , S. Demura , A. R. Derk , A. Dunsworth , D. Eppens , C. Erickson , et al., Nature 2022, 601, 531. 34847568 10.1038/s41586-021-04257-w PMC8791837

[8] K. J. Satzinger , Y.‐J. Liu , A. Smith , C. Knapp , M. Newman , C. Jones , Z. Chen , C. Quintana , X. Mi , A. Dunsworth , C. Gidney , I. Aleiner , F. Arute , K. Arya , J. Atalaya , R. Babbush , J. C. Bardin , R. Barends , J. Basso , A. Bengtsson , A. Bilmes , M. Broughton , B. B. Buckley , D. A. Buell , B. Burkett , N. Bushnell , B. Chiaro , R. Collins , W. Courtney , S. Demura , et al., Science 2021, 374, 1237. 34855491 10.1126/science.abi8378

[9] T. I. Andersen , Y. D. Lensky , K. Kechedzhi , I. Drozdov , A. Bengtsson , S. Hong , A. Morvan , X. Mi , A. Opremcak , R. Acharya , R. Allen , M. Ansmann , F. Arute , K. Arya , A. Asfaw , J. Atalaya , R. Babbush , D. Bacon , J. C. Bardin , G. Bortoli , A. Bourassa , J. Bovaird , L. Brill , M. Broughton , B. B. Buckley , D. A. Buell , T. Burger , B. Burkett , N. Bushnell , Z. Chen , et al., (Preprint) arXiv:2210.10255, submitted: Oct 2022.

[10] X. Mi , M. Sonner , M. Y. Niu , K. W. Lee , B. Foxen , R. Acharya , I. Aleiner , T. I. Andersen , F. Arute , K. Arya , A. Asfaw , J. Atalaya , R. Babbush , D. Bacon , J. C. Bardin , J. Basso , A. Bengtsson , G. Bortoli , A. Bourassa , L. Brill , M. Broughton , B. B. Buckley , D. A. Buell , B. Burkett , N. Bushnell , Z. Chen , B. Chiaro , R. Collins , P. Conner , W. Courtney , et al., Science 2022, 378, 785. 36395220 10.1126/science.abq5769

[11] F. Arute , K. Arya , R. Babbush , D. Bacon , J. C. Bardin , R. Barends , R. Biswas , S. Boixo , F. G. S. L. Brandao , D. A. Buell , B. Burkett , Y. Chen , Z. Chen , B. Chiaro , R. Collins , W. Courtney , A. Dunsworth , E. Farhi , B. Foxen , A. Fowler , C. Gidney , M. Giustina , R. Graff , K. Guerin , S. Habegger , M. P. Harrigan , M. J. Hartmann , A. Ho , M. Hoffmann , T. Huang , et al., Nature 2019, 574, 505. 31645734 10.1038/s41586-019-1666-5

[12] A. P. M. Place , L. V. H. Rodgers , P. Mundada , B. M. Smitham , M. Fitzpatrick , Z. Leng , A. Premkumar , J. Bryon , A. Vrajitoarea , S. Sussman , G. Cheng , T. Madhavan , H. K. Babla , X. H. Le , Y. Gang , B. Jäck , A. Gyenis , N. Yao , R. J. Cava , N. P. de Leon , A. A. Houck , Nat. Commun. 2021, 12, 1779. 33741989 10.1038/s41467-021-22030-5 PMC7979772

[13] C. Wang , X. Li , H. Xu , Z. Li , J. Wang , Z. Yang , Z. Mi , X. Liang , T. Su , C. Yang , G. Wang , W. Wang , Y. Li , M. Chen , C. Li , K. Linghu , J. Han , Y. Zhang , Y. Feng , Y. Song , T. Ma , J. Zhang , R. Wang , P. Zhao , W. Liu , G. Xue , Y. Jin , H. Yu , npj Quantum Inf. 2022, 8, 1.

[14] A. P. Read , B. J. Chapman , C. U. Lei , J. C. Curtis , S. Ganjam , L. Krayzman , L. Frunzio , R. J. Schoelkopf , Phys. Rev. Applied 2023, 19, 034064.

[15] C. Wang , C. Axline , Y. Y. Gao , T. Brecht , Y. Chu , L. Frunzio , M. H. Devoret , R. J. Schoelkopf , Appl. Phys. Lett. 2015, 107, 162601.

[16] D. P. Lozano , M. Mongillo , X. Piao , S. Couet , D. Wan , Y. Canvel , A. M. Vadiraj , T. Ivanov , J. Verjauw , R. Acharya , J. Van Damme , F. A. Mohiyaddin , J. Jussot , P. P. Gowda , A. Pacco , B. Raes , J. Van de Vondel , I. P. Radu , B. Govoreanu , J. Swerts , A. Potočnik , K. De Greve , (Preprint) arXiv:2211.16437, submitted: Nov 2022.

[17] D. W. Face , D. E. Prober , W. R. McGrath , P. L. Richards , Appl. Phys. Lett. 1986, 48, 1098.

[18] D. W. Face , D. E. Prober , J. Vac. Sci. Technol., A 1987, 5, 3408.

[19] C. Müller , J. H. Cole , J. Lisenfeld , Rep. Prog. Phys. 2019, 82, 124501. 31404914 10.1088/1361-6633/ab3a7e

[20] N. P. de Leon , K. M. Itoh , D. Kim , K. K. Mehta , T. E. Northup , H. Paik , B. S. Palmer , N. Samarth , S. Sangtawesin , D. W. Steuerman , Science 2021, 372, eabb2823. 33859004 10.1126/science.abb2823

[21] K. D. Crowley , R. A. McLellan , A. Dutta , N. Shumiya , A. P. M. Place , X. H. Le , Y. Gang , T. Madhavan , N. Khedkar , Y. C. Feng , E. A. Umbarkar , X. Gui , L. V. H. Rodgers , Y. Jia , M. M. Feldman , S. A. Lyon , M. Liu , R. J. Cava , A. A. Houck , N. P. de Leon , Disentangling Losses in Tantalum Superconducting Circuits, (Preprint) arXiv:2301.07848, submitted: Jan 2023.

[22] A. Premkumar , C. Weiland , S. Hwang , B. Jäck , A. P. M. Place , I. Waluyo , A. Hunt , V. Bisogni , J. Pelliciari , A. Barbour , M. S. Miller , P. Russo , F. Camino , K. Kisslinger , X. Tong , M. S. Hybertsen , A. A. Houck , I. Jarrige , Commun. Mater. 2021, 2, 72.

[23] J. F. Moulder , Handbook of X‐ray Photoelectron Spectroscopy: A Reference Book of Standard Spectra for Identification and Interpretation of XPS Data, Physical Electronics Division, Perkin‐Elmer Corporation, Waltham, MA, USA 1992.

[24] P. M. Sherwood , Surf. Interface Anal. 2019, 51, 589.

[25] M. H. Engelhard , D. R. Baer , A. Herrera‐Gomez , P. M. A. Sherwood , J. Vac. Sci. Technol. A 2020, 38, 063203.

[26] R. Pugliese , G. Paolucci , https://vuo.elettra.eu/services/elements/WebElements.html, last accessed on September 2022.

[27] F. J. Himpsel , J. F. Morar , F. R. McFeely , R. A. Pollak , G. Hollinger , Phys. Rev. B 1984, 30, 7236.

[28] A. Jablonski , C. J. Powell , J. Phys. Chem. Ref. Data 2020, 49, 033102.

[29] B. Henke , E. Gullikson , J. Davis , At. Data Nucl. Data Tables 1993, 54, 181.

[30] A. Jablonski , C. J. Powell , J. Phys. Chem. Ref. Data 2020, 49, 033102.

[31] H. Shinotsuka , S. Tanuma , C. J. Powell , D. R. Penn , Surf. Interface Anal. 2015, 47, 871. 10.1002/sia.6123 PMC5524379 28751796

[32] J. Verjauw , A. Potočnik , M. Mongillo , R. Acharya , F. Mohiyaddin , G. Simion , A. Pacco , T. Ivanov , D. Wan , A. Vanleenhove , L. Souriau , J. Jussot , A. Thiam , J. Swerts , X. Piao , S. Couet , M. Heyns , B. Govoreanu , I. Radu , Phys. Rev. Appl. 2021, 16, 014018.

