# Supplementary material for: Chemical Profiles of the Oxides on Tantalum in State of the Art Superconducting Circuits
Source: Adv Sci (Weinh). 2023 May 11;10(21):2300921. doi: 10.1002/advs.202300921 (PMC10375100; doi:10.1002/advs.202300921)
Supplement: Supplementary file 1 — Supporting Information [file ADVS-10-2300921-s001.pdf]

ADVANCED SCIENCE
Open Access

Supporting Information

for *Adv. Sci.*, DOI 10.1002/advs.202300921

Chemical Profiles of the Oxides on Tantalum in State of the Art Superconducting Circuits

Russell A. McLellan, Aveek Dutta, Chenyu Zhou, Yichen Jia, Conan Weiland, Xin Gui, Alexander P. M. Place, Kevin D. Crowley, Xuan Hoang Le, Trisha Madhavan, Youqi Gang, Lukas Baker, Ashley R. Head, Iradwikanari Waluyo, Ruoshui Li, Kim Kisslinger, Adrian Hunt, Ignace Jarrige, Stephen A. Lyon, Andi M. Barbour, Robert J. Cava, Andrew A. Houck, Steven L. Hulbert, Mingzhao Liu, Andrew L. Walter* and Nathalie P. de Leon**

Supporting Information for: Chemical profiles of the oxides on tantalum in state of the art superconducting circuits

Russell A. McLellan† *Aveek Dutta*† *Chenyu Zhou* *Yichen Jia* *Conan Weiland* *Xin Gui* *Alexander P. M. Place* *Kevin D. Crowley* *Xuan Hoang Le* *Trisha Madhavan* *Youqi Gang* *Lukas Baker* *Ashley R. Head* *Iradwikanari Waluyo* *Ruoshui Li* *Kim Kisslinger* *Adrian Hunt* *Ignace Jarrige* *Stephen A. Lyon* *Andi M. Barbour* *Robert J. Cava* *Andrew A. Houck* *Steven L. Hulbert* *Mingzhao Liu** *Andrew L. Walter** *Nathalie P. de Leon**
†These authors contributed equally.

S1 Materials

All samples used in the variable energy X-ray photoelectron spectroscopy (VEXPS) measurements were approximately 200 nm thick α-Ta(111). Measurements reported in the main text were performed on a film deposited by DC magnetron sputtering onto c-plane sapphire by Star Cryoelectronics. Measurements reported in Section S7 were performed on a film deposited by DC magnetron sputtering onto c-plane sapphire at Princeton University. Phase and orientation of both tantalum films were confirmed by X-ray diffraction and measurements of both the superconducting critical temperature and critical magnetic field in a Physical Property Measurement System. All samples were approximately 7 mm x 4 mm rectangles. Morphology differences between the two films are described in Section S8.

S2 Methods

10:1 buffered oxide etch (BOE) is a mixture of 10 parts 40% NH_4F solution to 1 part 49% HF solution by volume. We procured 10:1 BOE from Transene. BOE treated samples were placed in buffered oxide etch at room temperature and were not agitated. After 20 minutes, the samples were removed and triple rinsed in de-ionized water and 2-propanol before being blown dry in N_2.

The triacid treatment is 1:1:1 equal mix by volume of 95-98% H_2SO_4, 70% HNO_3, and 70% $HClO_4$ solutions (all percentages by weight). We procured all solutions from SigmaAldrich (catalogue numbers: H_2SO_4 - 258105, HNO_3 - 225711, $HClO_4$ - 244252). After the sample was added to the mixture, it was heated to 200 °C for 2 hours and then allowed to cool for 1 hour. During this process, the exhaust gas was cooled and bubbled through water. No agitation was performed. After cooling, the sample was removed, triply rinsed in de-ionized water and 2-propanol before being blown dry in N_2.

All films were treated in piranha solution for 20 minutes. BOE and triacid treated samples were treated in piranha solution prior to undergoing BOE or triacid treatments. Native samples were treated several hours before being inserted into the vacuum chamber for VEXPS. Piranha solution was prepared with 2 parts H_2SO_4 to 1 part H_2O_2 by volume, initially at room temperature. No external heating or agitation was performed. After being removed from the piranha solution, samples were triply rinsed in de-ionized water and 2-propanol before being blown dry in N_2. The effect of the piranha treatment is explored in Section S6.

VEXPS measurements were performed at the Spectroscopy Soft and Tender-1 and Spectroscopy Soft and Tender-2 (SST-1 and SST-2) beam lines at the National Synchrotron Light Source II at Brookhaven National Laboratory. SST-1 was used for X-ray energies less than 2000 eV and SST-2 was used for X-ray energies greater than or equal to 2000 eV. The difference between SST-1 and SST-2 is in the energy range of the X-ray beam sent to the sample; beam lines share the

same vacuum chamber. The step size for VEXPS measurements was 0.05 eV and dwell time was 100 ms. The detector pass energy was varied from 20 eV to 200 eV as X-ray energy was changed based on the observed electron counts and whether we could resolve the Ta^0_{int} shoulder peak. Depending on the experiment, a separate sample of either silver or gold was scanned at each X-ray energy as a binding energy reference. When a silver reference was used, we set the binding energy of the $Ag3d_{5/2}$ peak to 368.3 eV. When a gold reference was used, we set the binding energy of the $Au4f_{7/2}$ peak to 84 eV.

S3 XPS trace analysis

S3.1 Uncertainty calibration of XPS data

The number of electron counts varies significantly from datapoint to datapoint. The maximal number of counts for different spectra can vary by over two orders of magnitude across the X-ray energy we scanned. This difference is largely attributed to differences in incident X-ray photon flux. We expect the uncertainties in our measured photoelectron intensity to be a function of the number of counts, and we need to calibrate the uncertainties to ensure that we are fitting the peaks correctly.

To calibrate our error bars, we fit a line to regions of traces that contain no peaks. The Ta4f peaks are close in binding energy to the Ta5p and Ta5s peaks, and therefore it was not possible to find a region near the Ta4f peaks that contained only background counts. However, each time we measured Ta4f traces at a particular X-ray energy, we also measured the O1s and C1s peaks on each tantalum sample. We also have Ag3d spectra on the reference sample that we used to calibrate the binding energy at each photon energy. We set our binding energy range on these peaks large enough to capture a region several eV wide with no observable satellite loss peaks. As there are no observable peaks in these regions, we assume that a line is the best fit to each dataset.

We initially assume that the electron count statistics are Poissonian, and therefore $\sigma_{I_i} = \sqrt{I_i}$, where I_i is the number of counts for the ith datapoint and σ_x is the uncertainty in the measurement x. For each C1s, O1s, and Ag3d trace, we fit a line to background regions using these Poissonian error bars, and then scale the uncertainties so that the reduced χ^2 value of the fit is unity. The uncertainties are now given by $\sigma_{I_i} = \alpha\sqrt{I_i}$, where α is a scalar. The binding energy regions we considered as the background are shown in Table S1. The uncertainty scaling is shown for an example C1s trace in Figure S1(a).

A value of α was fit individually to each O1s, C1s, and Ag3d trace. There is no systematic trend with the value of α versus either the mean electron kinetic energy of the scan or the mean number of electron counts for the scan (Figure S1(c-d)). All values of α appear to be drawn from a unimodal distribution with mean $\overline{\alpha} = 6.4 \pm 0.9$ (Figure S1(b)). Based on these results, we use error bars $\sigma_{I_i} = \overline{\alpha}\sqrt{I_i}$ for all of our data.

We interpret the value of $\overline{\alpha}$ being larger than unity as consistent with the large amount of gain in the electron detection system. We also note that any overall scaling factor $\overline{\alpha}$ would not affect the fits of the XPS peaks, but only the reduced χ^2 value. The parameters resulting from our fits would be different only if the uncertainties scaled in a manner other than $\sigma_{I_i} \propto \sqrt{I_i}$.

Table S1: Binding energy regions considered empty for uncertainty calibration.

	O1s	C1s	Ag3d
Lower bound (eV)	538	291	360
Upper bound (eV)	543	295	363

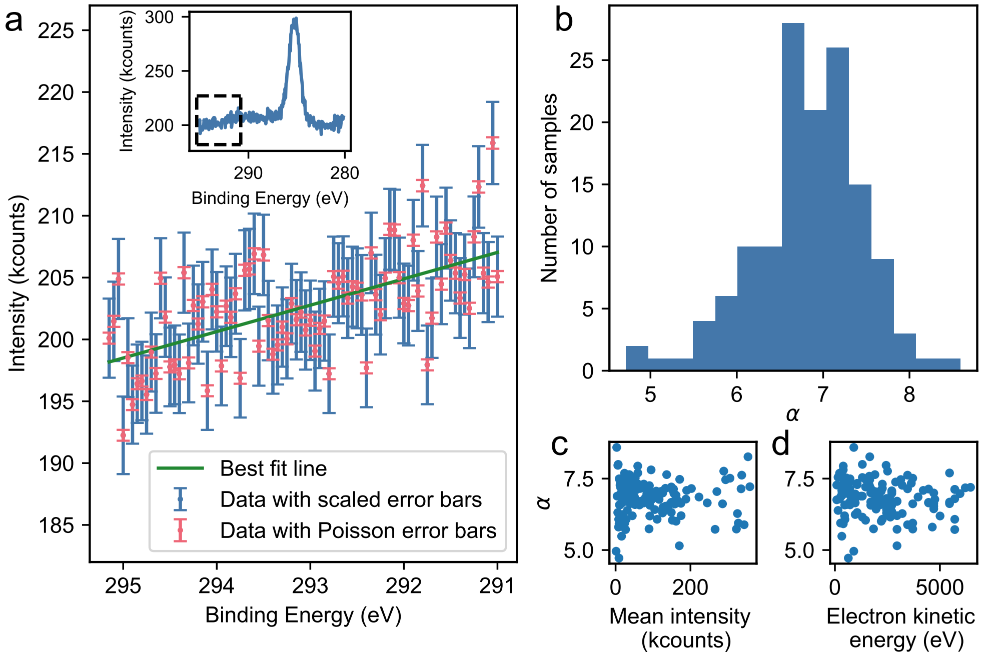

Figure S1: a) Photoelectron counts for a C1s scan between 291 eV and 295 eV at an incident X-ray energy of 2000 eV. We observe no loss peaks in this region. Data is plotted in red with errors bars assuming a Poisson distribution. However, the best fit line to the data (green) does not give a reduced χ^2 of unity with Poisson error bars. The errors bars must be scaled by a number α to give $\chi^2 = 1$. These scaled error bars are plotted in blue. (Inset: The full C1s scan with the area in the main plot indicated) b) Histogram of the scaling parameter α obtained by scaling error bars in empty regions of all C1s, O1s, and Ag3d regions as in a). The mean value of α is $\overline{\alpha} = 6.4 \pm 0.9$. c-d) the data in b) plotted versus the mean intensity (c) and electron kinetic energy (d) of each scan. There is no systematic trend with either variable, indicating that a single scaling factor $\overline{\alpha}$ is sufficient to capture the uncertainty in the intensity in all scans.

S3.2 Background subtraction

We subtracted a Shirley background from all Ta4f and O1s XPS spectra before fitting. The Shirley background attempts to correct for the step change in background signal seen before and after a large peak, attributed to electrons from the peak which are scattered before being detected at a lower kinetic energy. The initial presentation of the Shirley background is given in [1] and it is discussed in more detail in [2]. The Shirley background correction process is presented here based on those treatments, and we include a method of propagating error through the background correction.

First, we choose binding energies above and below the peaks of interest where we assume that any signal present is only background signal. The intensity of electrons at these binding energies will be used in our background subtraction, so we average over a small number of binding energy points to extract a mean and uncertainty of the background counts.

We subtract a flat background, $\overline{I_1}$, from the spectrum, equal to the mean number of counts at the lower binding energy. This initial flat background correction compensates for any constant source of noise that is independent of the electrons from the peaks. The intensity after the flat background correction is given by:

$$I_i^{'} = I_i - \overline{I_1}, \tag{1}$$

where $I_i^{'}$ is the flat-background corrected intensity at index i, and I_i is the uncorrected intensity at binding energy index i. The uncertainty from the flat background is propagated to the uncertainty in the flat-background corrected intensities by:

$$\sigma_{I_i^{'}}^2 = \sigma_{I_i}^2 + \sigma_{I_1}^2, \tag{2}$$

where σ_A indicates the uncertainty in the variable A.

After the flat background correction, we apply the Shirley background correction. The Shirley background is given by:

$$s_i = \left(\frac{\sum\limits_{j \le i} I_j^{'}}{\sum\limits_{k} I_k^{'}} \right) (I_f^{'} - I_1^{'}), \tag{3}$$

where s_i is the Shirley background at index i, and the indices run from 1 to f, where f is the index corresponding to the highest binding energy. The Shirley background corrected intensity is given by $I_i^{''} = I_i^{'} - s_i$. To calculate the uncertainty in the Shirley-corrected data, we use the following formula for error propagation [3]:

$$\sigma_B^2 = \sum_i \sigma_{b_i}^2 \left(\frac{\partial B}{\partial b_i} \right)^2, \tag{4}$$

where B is a function of the b_is and each b_i has a known uncertainty. We have assumed that all σ_{b_i} are independent in Equation 4. Applying Equation 4 to $I_i^{''}$, we find:

$$\sigma_{I_i^{''}}^2 = \sigma_{I_i^{'}}^2 + g_i^2 (I_f^{'} - I_1^{'})^2 \left(\frac{\sigma_{g_i}^2}{g_i^2} + \frac{\sigma_{I_f^{'}}^2 + \sigma_{I_1^{'}}^2}{(I_f^{'} + I_1^{'})^2} \right), \tag{5}$$

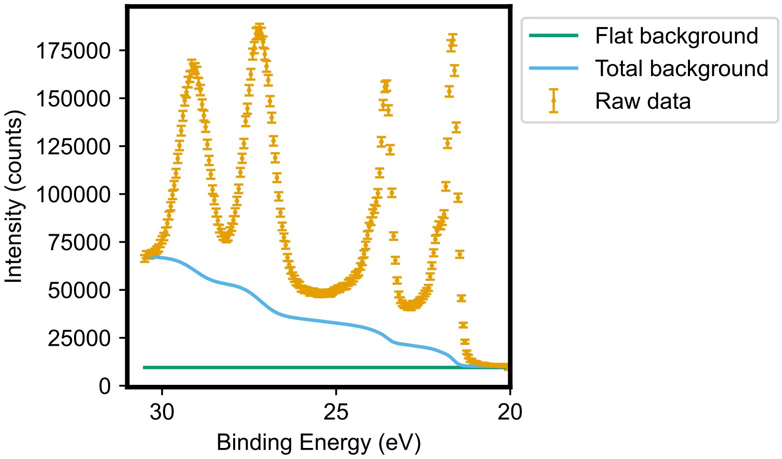

Figure S2: Data and calculated background for the Ta4f spectra measured on the untreated ("Native") sample with an X-ray energy of 2200 eV. Both flat and total (flat + Shirley) backgrounds are shown. The flat background corrects for any constant noise unrelated to the electrons from the peaks, while the Shirley background corrects for electrons from the peaks which are scattered before being collected.

where:

$$g_i = \frac{\sum_{j\leq i} I'_j}{\sum_k I'_k} \tag{6}$$

$$\sigma^2_{g_i} = \left(\frac{1-g_i}{\sum_k I'_k}\right)^2 \sum_{j\leq i} \sigma^2_{I'_j} + \left(\frac{g_i}{\sum_k I'_k}\right)^2 \sum_{j>i} \sigma^2_{I'_j}. \tag{7}$$

We perform background subtraction for both Ta4f and O1s peaks. For Ta4f peaks, the binding energy range for background subtraction is 20 eV to 30.5 eV. For O1s peaks, the binding energy range for background subtraction is 528 eV to 536 eV. An example of background subtraction on a Ta4f spectrum is shown in Figure S2.

S3.3 XPS peak fitting with constraints

As described in the main text, XPS peaks are fit with either Gaussian (Ta^{5+}, Ta^{3+}, Ta^{1+}, and O2s), or skewed Voigt (Ta^0 and Ta^0_{int}) profiles [2]. Each Ta peak is doubled due to the strong spin orbit coupling of tantalum, leaving us with a total of 11 peaks per Ta4f spectrum. In order to fit these peaks with minimal uncertainty, we fit all Ta4f spectra for each sample simultaneously, constraining peak locations, amplitudes, widths, and skewnesses as described in this section. Our peak fitting is implemented with the lmfit Python package [4].

A Gaussian profile is described by:

$$f(x; A, \mu, \sigma) = \frac{A}{\sigma\sqrt{2\pi}} e^{-(x-\mu)^2/2\sigma^2}, \tag{8}$$

where A is the area under the curve, μ is the center of the profile, and σ is the width of the profile. A skewed Voigt profile is described by:

$$f(x; A, \mu, \sigma, \lambda) = \frac{A\text{Re}[w(z)]}{\sigma\sqrt{2\pi}} \left(1 + \text{erf}\left[\frac{\lambda(x-\mu)}{\sigma\sqrt{2}}\right]\right), \tag{9}$$

where A, μ, and σ have the same meanings as in Equation 8, λ is the dimensionless skewness parameter, erf is the error function, and z and $w(z)$ are given by:

$$z = \frac{x - \mu - i\sigma}{\sigma\sqrt{2}} \tag{10}$$

$$w(z) = e^{-z^2}\mathrm{erfc}(-iz). \tag{11}$$

In Equation 11, $\mathrm{erfc}(-iz)$ = 1-$\mathrm{erf}(-iz)$ is the complementary error function.

Physically, for each peak, A represents the total photoelectron intensity, μ represents the binding energy of the peak, σ characterizes broadening (including broadening introduced by the photoelectron detector), and λ characterizes the shake-up satellite structure of conductive compounds. We use these physical interpretations to constrain parameters as follows.

First, we assume that the shake-up satellite structure of the Ta^0_{int} and Ta^0 states are identical and unchanged across experiments at different X-ray energies. This assumption implies that a single value of λ can be used for all Ta^0_{int} and Ta^0 peaks across all X-ray energies for each sample.

Second, each pair of peaks that arise from the $Ta_{7/2}$ and $Ta_{5/2}$ spin states of the same tantalum oxidation state should share the same σ parameter. Further, we assume that the proportion of electron population in the $Ta_{7/2}$ and $Ta_{5/2}$ states, the proportion of the X-ray cross sections of the $Ta_{7/2}$ and $Ta_{5/2}$ states, and spin-orbit coupling strengths are independent of the tantalum oxidation state and incident X-ray energy. These assumptions imply that the ratio of A and the difference in μ between any pair of $Ta_{7/2}$ and $Ta_{5/2}$ peaks is the same

Third, we assume that the binding energy of each peak does not change between experiment at different X-ray energies. In practice, variations on the order of 0.1 eV are observed in the positions of the Ta^{5+} and Ta^0 peaks, which we attribute to charging of the tantalum oxide layer. Instead of fixing binding energy positions absolutely, we constrain relative peak position. We allow the binding energy of the Ta^{5+} and Ta^0 peaks to vary at each X-ray energy, as these peaks are easily located. The positions of the Ta^{1+} and Ta^{3+} are constrained relative to the position of the Ta^{5+} peak across all X-ray energies, and likewise the position of the Ta^0_{int} peak is constrained relative to the Ta^0 peak. We constrain the O2s peak to have a single binding energy, although in practice, this peak is small and found to be quite wide, so variations in the O2s binding energy are unlikely to have a significant impact on the fit.

Fourth, we assume that for a given XPS spectrum, the broadening of the Ta^0 and Ta^0_{int} peaks are the same, as is the broadening of the Ta^{1+}, Ta^{3+}, and Ta^{5+} peaks. We are assuming that only three different values of σ are needed for each Ta4f spectrum; one for the tantalum oxide species, one for the metallic species, and one for the O2s peak. In practice, relaxing this assumption does not significantly affect the peak fits.

Fifth, we constrain the amplitude of the O2s peak to 5% of the amplitude of the O1s spectrum. The X-ray cross-section for the O1s and O2s states are approximately constant in the X-ray energy range 600 eV to 1500 eV [5], and we extrapolate this ratio out to our maximum X-ray energy of 7000 eV. The intensity of the O2s peak is typically less than 5% of the Ta4f intensity, so we do not believe that this extrapolation introduces significant error. Note that we are neglecting the kinetic energy difference between an O1s photoelectron and an O2s photoelectron. At each X-ray energy, we numerically integrate the area under a background corrected O1s spectrum between binding energies 528 eV and 536 eV to calculate the O1s intensity, and this value is scaled to fix the O2s energy at the corresponding Ta4f spectrum.

With these constraints in place, for our native tantalum dataset with 17 different X-ray energies, we have 193 free parameters to fit 187 different peaks. This reduction is a significant improvement over the naive method where each parameter is independent, which requires 340 free

parameters for the same 187 peaks. The full results of this fitting method are shown for the native tantalum (Figure S3), BOE treated tantalum (Figure S4), and triacid treated tantalum (Figure S5).

S3.4 Propagation of error to photoelectron intensity fractions

The fitted parameters from the XPS spectra which are used in the depth profile analysis are the intensity fractions of each of the $Ta_{7/2}$ peaks,

$$W_n = \frac{A_n}{\sum\limits_m A_m}, \tag{12}$$

where W_n is the intensity fraction of the oxidation state n, A_n is the intensity from the oxidation state n, and the index n belongs to the set $\{Ta^0, Ta^0_{\text{int}}, Ta^{1+}, Ta^{3+}, Ta^{5+}\}$. In addition to f_n, we will also need σ_{f_n}, the uncertainty in the intensity fraction.

We propagate the uncertainty to the intensity fraction using the following formula [3]:

$$\sigma_B^2 = \sum_{i,j} \sigma_{b_i b_j}^2 \left(\frac{\partial B}{\partial b_i}\right)\left(\frac{\partial B}{\partial b_j}\right), \tag{13}$$

where B is a function of the b_is, and $\sigma_{b_i b_j}^2$ is the covariance between b_i and b_j, and $\sigma_{b_i b_i}^2 = \sigma_{b_i}^2$ is the variance of b_i. In Equation 13, we have not assumed that the errors are uncorrelated, as the different intensities do correlate with each other.

The empirical covariance matrix is calculated and reported by the lmfit Python module in addition to the fitted parameters. We apply Equation 13 to Equation 12 to arrive at:

$$\sigma_{W_n}^2 = \sigma_{A_n}^2 \left(\frac{1 - W_n}{\sum\limits_m A_m}\right)^2 + \left(\frac{W_n}{\sum\limits_m A_m}\right)^2 \sum_{\ell \neq n} \sigma_{A_\ell}^2 - 2\frac{W_n(1 - W_n)}{\left(\sum\limits_m A_m\right)^2} \sum_{\ell \neq n} \sigma_{A_\ell A_n}^2, \tag{14}$$

which we use to set the uncertainties in the intensity fractions used when fitting a depth profile.

S4 Chemical depth profile analysis

S4.1 General modeling of the film

Here we model the object of study as a multicomponent thin film placed on a uniform substrate of infinite thickness. The object occupies the half-space of $x \geq 0$, and contains N unique species $(S_n, n = 1, ..., N)$ that are spatially mixed, including the substrate species (S_N). The mixing is inhomogenous along x but is homogenous along the other two dimensions. During mixing, we assume the volume of each species is conserved, so that a volume fraction profile $\{F_n(x)\}$ $(n = 1, ..., N)$ is defined for each depth x, with the total volume fraction constraint $\sum\limits_{n=1}^{N} F_n(x) = 1$ for all x. The volume fraction of the substrate species, F_N, follows the limiting behavior of $\lim_{x\to\infty} F_N(x) = 1$.

Now consider an atom of interest Q, that has a mass density of ρ_n within each species S_n. As such, the total mass density of all atoms Q at a depth of x is

$$\rho(x) = \sum_{n=1}^{N} \rho_n F_n(x). \tag{15}$$

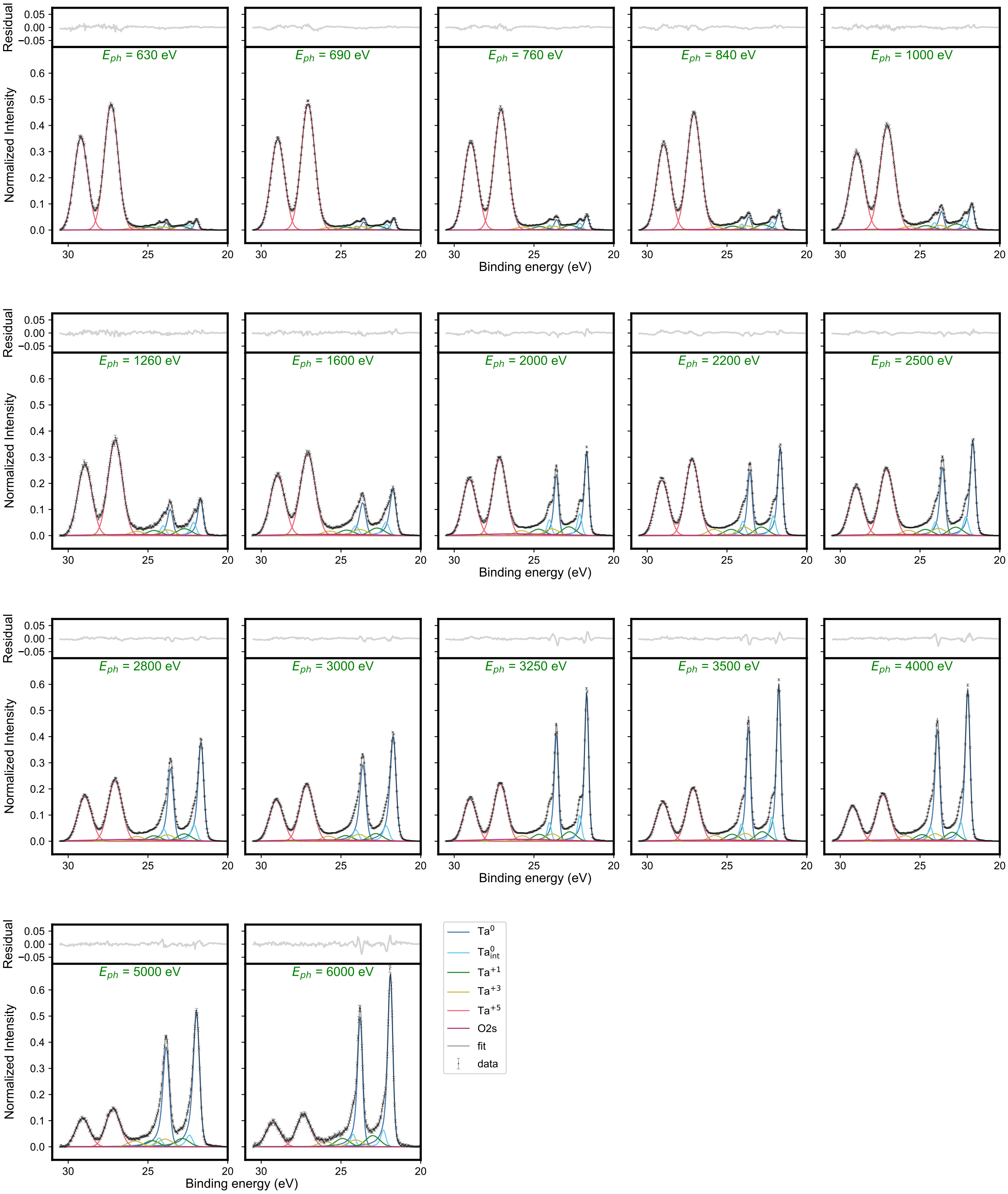

Figure S3: Fitted Ta4f intensity spectra for all X-ray energies on the untreated (”Native”) sample. All spectra are fitted simultaneously with certain parameters constrained between spectra, as described in the text. Three of these fitted spectra are shown in the main text in Figure 2.

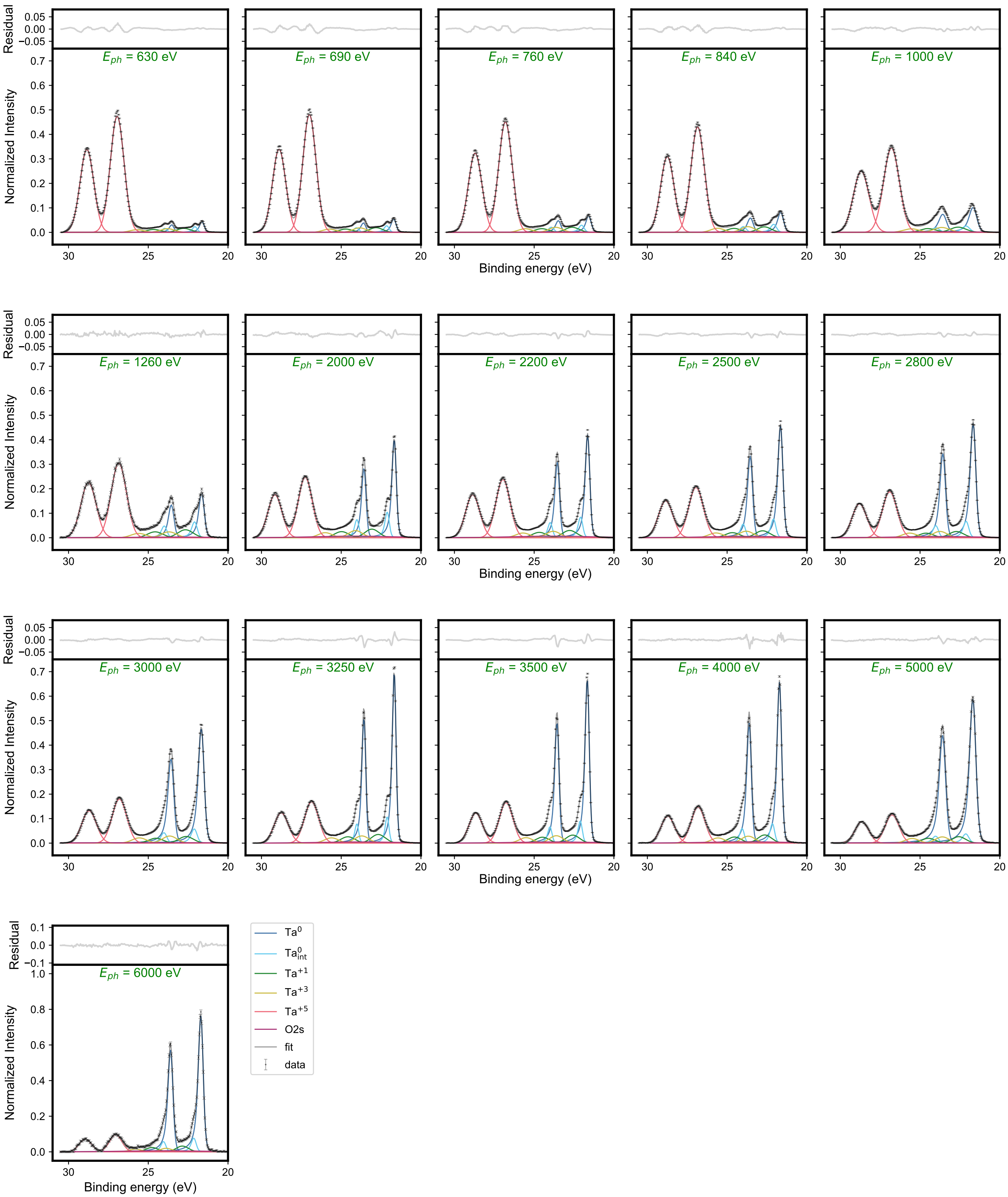

Figure S4: Fitted Ta4f intensity spectra for all X-ray energies on the BOE treated sample. All spectra are fitted simultaneously with certain parameters constrained between spectra, as described in the text. Note that the $E_{ph} = 6000$ eV plot has different y-axis limits from the other spectra.

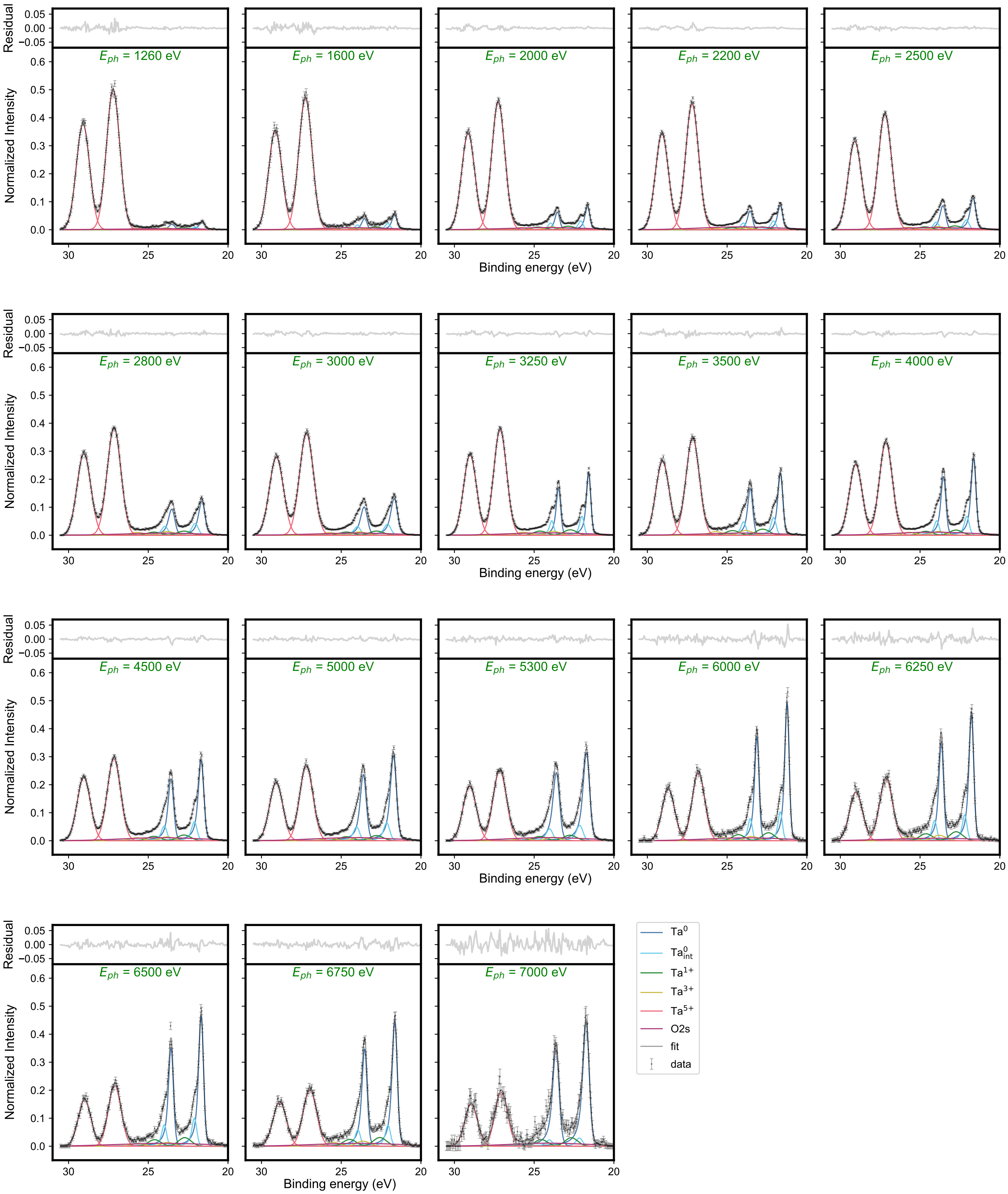

Figure S5: Fitted Ta4f intensity spectra for all X-ray energies on the triacid treated sample. All spectra are fitted simultaneously with certain parameters constrained between spectra, as described in the text.

S4.2 Photon and photoelectron attenuation

Consider a beam of incident photons that have photon energy E_{ph}, incident angle Θ_{ph}, and photon flux I_0 normalized to unity. The beam is attenuated across the thin film due to photon absorption by atom Q. Across a thin slab of thickness ds at a depth of s, the attenuation of beam flux can be written as

$$dI = -I(s, E_{\text{ph}}, \Theta_{\text{ph}}) \frac{\mu}{\rho_0} \frac{\rho(s)}{\sin \Theta_{\text{ph}}} ds, \tag{16}$$

in which the term μ/ρ_0 is the tabulated mass attenuation coefficient as a function of E_{ph}, i.e., $\mu/\rho_0 = \mu(E_{\text{ph}})/\rho_0$. Integration over Equation (16) produces photon flux at the depth x, as

$$I(x, E_{\text{ph}}, \Theta_{\text{ph}}) = \exp\left(-\int_0^x \frac{\mu(E_{\text{ph}})}{\rho_0} \frac{\rho(s)}{\sin \Theta_{\text{ph}}} ds\right). \tag{17}$$

For photoelectron generation, we assume an atom Q has a photoelectron yield η that is independent of its species assignment. As such, the species S_n contained within a thin slab of dx at a depth of x should give rise to a photoelectron generation rate dg_n as:

$$\begin{aligned} dg_n &= \eta \rho_n F_n(x) I(x, E_{\text{ph}}, \Theta_{\text{ph}}) dx \\ &= \eta \rho_n F_n(x) \exp\left(-\int_0^x \frac{\mu(E_{\text{ph}})}{\rho_0} \frac{\rho(s)}{\sin \Theta_{\text{ph}}} ds\right) dx. \end{aligned} \tag{18}$$

Photoelectrons generated from atoms Q in species S_n have kinetic energy $E_k = E_{\text{ph}} - E_{b,n}$, in which $E_{b,n}$ is the binding energy of the core level of Q in S_n. With a photoelectron collection angle Θ_{el}, photoelectrons generated at depth x travel through a distance of $x/\sin\Theta_{\text{el}}$ across the film, leading to an attenuation factor of $\exp\left(-x\lambda_{\text{el}}^{-1}(E_{\text{ph}} - E_{b,n})/\sin\Theta_{\text{el}}\right)$, in which $\lambda_{\text{el}}(E_{\text{ph}} - E_{b,n})$ is the inelastic mean free path of a photoelectron at the energy $E_{\text{ph}} - E_{b,n}$. As such, the species S_n contained within a thin slab of dx at a depth of x will contribute

$$\begin{aligned} dA_n &= \gamma dg_n \exp\left(-\frac{x}{\lambda_{\text{el}}(E_{\text{ph}} - E_{b,n}) \sin \Theta_{\text{el}}}\right) \\ &= \gamma \eta \rho_n F_n(x) \exp\left(-\int_0^x \frac{\mu(E_{\text{ph}})}{\rho_0} \frac{\rho(s)}{\sin \Theta_{\text{ph}}} ds - \frac{x}{\lambda_{\text{el}}(E_{\text{ph}} - E_{b,n}) \sin \Theta_{\text{el}}}\right) dx \\ &= \gamma \eta \rho_n F_n(x) T_n(x, E_{\text{ph}}, \Theta_{\text{ph}}, \Theta_{\text{el}}) dx \end{aligned} \tag{19}$$

to the final photoelectron spectrum, where γ is the photoelectron collection efficiency and $T_n(x, E_{\text{ph}}, \Theta_{\text{ph}}, \Theta_{\text{el}})$ covers the exponential attenuation term in the second line. It should be noted that the latter term only has very weak dependence on n, as the variance of $E_{b,n}$ has little relative influence on photoelectron kinetic energy $E_{\text{el}} - E_{b,n}$. As such, we may safely replace $T_n(x, E_{\text{ph}}, \Theta_{\text{ph}}, \Theta_{\text{el}})$ with a species-independent attenuation $T(x, E_{\text{ph}}, E_b, \Theta_{\text{ph}}, \Theta_{\text{el}})$, in which $E_b \simeq \langle E_{b,n} \rangle$ is the typical binding energy.

Integration over x produces the total contribution of species S_n to the final spectrum, as

$$A_n(E_{\text{ph}}, E_b, \Theta_{\text{ph}}, \Theta_{\text{el}}) = \gamma \eta \rho_n \int_0^\infty F_n(x) T(x, E_{\text{ph}}, E_b, \Theta_{\text{ph}}, \Theta_{\text{el}}) dx. \tag{20}$$

Finally, the species S_n contributes a normalized weight of

$$W_n(E_{\text{ph}}, E_b, \Theta_{\text{ph}}, \Theta_{\text{el}}) = \frac{\rho_n \int_0^\infty F_n(x) T(x, E_{\text{ph}}, E_b, \Theta_{\text{ph}}, \Theta_{\text{el}}) dx}{\sum_{n=1}^N \rho_n \int_0^\infty F_n(x) T(x, E_{\text{ph}}, E_b, \Theta_{\text{ph}}, \Theta_{\text{el}}) dx}, \tag{21}$$

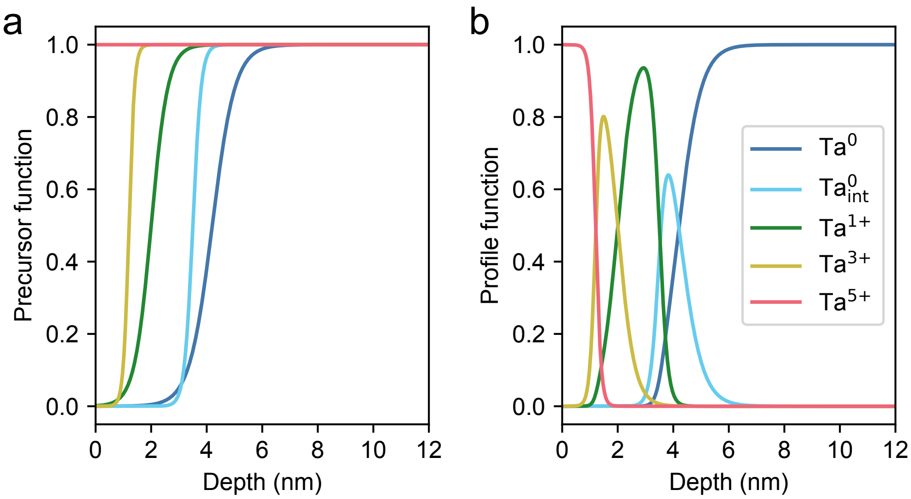

Figure S6: Example precursor function set (a) and corresponding profile function set (b). The precursor functions are sigmoids or a constant as described in Equation 22, and the profile functions are calculated from the precursors according to Equation 23. The profile function set has the property that at any depth, the sum of all functions is unity.

which directly corresponds to the results obtained by analyzing experimental XPS spectra. The fitting process will use experimentally obtained $\{W_n(E_{\mathrm{ph}}, E_b, \Theta_{\mathrm{ph}}, \Theta_{\mathrm{el}})\}$ to infer volume fractions $\{F_n(x)\}$.

S4.3 Basis set of volume fractions

A basis set is needed for volume fractions $\{F_n(x)\}$, so that a small number of fitting parameters can be used to model the spatial distribution of each species within the thin film. The basis set we use is generated with the following procedure. First, a set of N *precursor functions* are defined as

$$f_n(x) = \begin{cases} H(x) & (n = 1) \\ \left[1 + \exp\left(\frac{d_n - x}{w_n}\right)\right]^{-1} & (1 < n \leq N), \end{cases} \tag{22}$$

in which $H(x)$ is the unit step function, and d_n and w_n are center and width parameters for $f_n(x)$. Using the precursor functions, the volume fraction functions are generated as

$$F_n(x) = \begin{cases} (1 - f_{n+1}(x)) \prod_{i=1}^{n} f_i(x) & (1 \leq n < N) \\ \prod_{i=1}^{N} f_i(x) & (n = N). \end{cases} \tag{23}$$

Equation 23 will generate functions which automatically follow the sum rule $\sum_{n=1}^{N} F_n(x) = 1$. A typical initial guess of precursor functions $\{f_n(x)\}$ and generated volume fractions $\{F_n(x)\}$ are shown in Fig. S6. The basis set for the N species has a total of $2(N-1)$ fitting parameters ($N-1$ d_n's and $N-1$ w_n). Note that the precursor function $f_1(x)$ requires no parameters. With $N = 5$ unique species, the fitting procedure involves 8 fitting parameters.

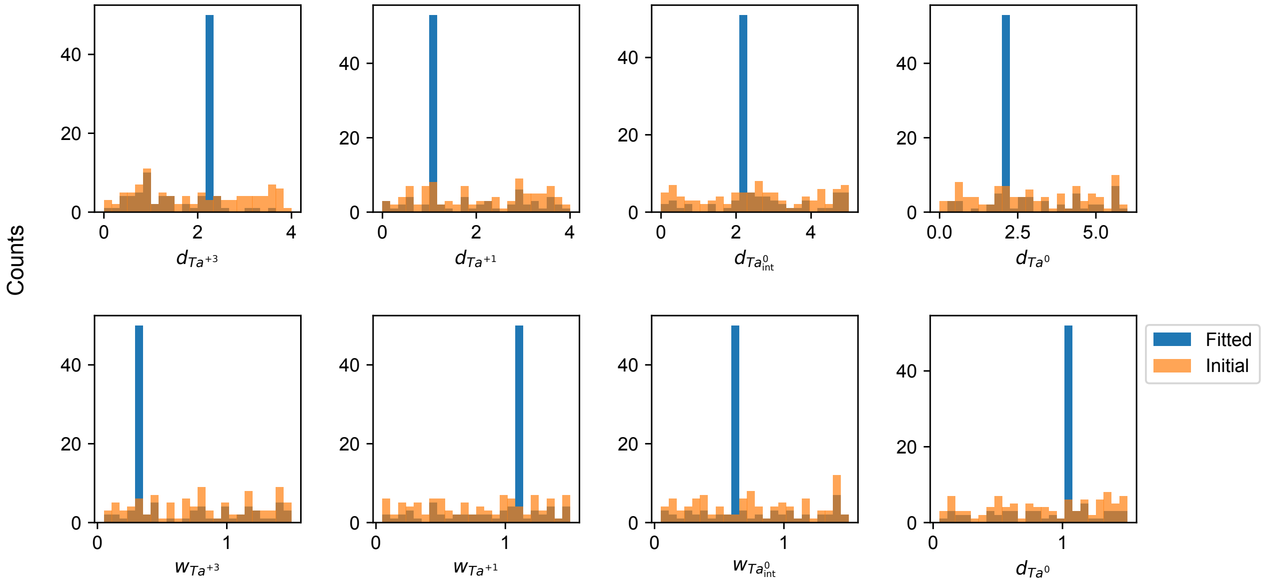

Figure S7: Initial and final parameters from 100 fits to the native oxide sample. Initial conditions for each fit were drawn from uncorrelated uniform distributions with bounds given in Table S2. Parameters are those specified in Equation 22, in units of nm, with numeric subscripts $\{1, 2, 3, 4\}$ replaced by the descriptive subscripts $Ta^{5+}, Ta^{3+}, Ta^{1+}\{Ta^0_{int}\}$

S4.4 Method to ensure a best fit

Fitting the model function, Equation 21, with the depth profile parameterizations, described in Section S4.3, to experimental data is a non-convex optimization problem. A fitting algorithm can terminate in a local minimum of the least squared objective function instead of a global minimum. Unlike the fits to XPS spectra described in Section S3, we may not have sufficient physical insight into our system to recognize and discard solutions corresponding to these local minima when we fit chemical depth profiles.

To overcome this issue, we perform 100 fits to the data from each sample, varying the initial conditions for each fit. For each fit, we draw initial conditions for the 8 parameters (4 d_ns and 4 w_ns specified in Equation 22) from uncorrelated uniform distributions. The bounds for these uniform distributions are given in Table S2; the bounds for the triacid treated sample were extended to account for the thicker oxide layer. For each sample we fit, we found that a large fraction of the 100 fits converged to the same set of final parameters. These sets of fitted parameters correspond to the lowest χ^2 value, and therefore we are confident that we have found the best chemical depth profile fit given our parameterization. Histograms of the initial and final parameter values for all 100 fits to the data taken from the native sample are shown in Figure S7.

Table S2: Uniform distribution bounds used for the initial conditions of depth profile fits. Parameters are the d_ns and w_ns from Equation 22 with numeric subscripts $\{2, 3, 4, 5\}$ replaced by the descriptive subscripts $Ta^{3+}, Ta^{1+}, \{Ta^0_{int}, Ta^0\}$. All values are given in nm.

Film	$d_{Ta^{3+}}$	$d_{Ta^{1+}}$	$d_{Ta^0_{int}}$	d_{Ta^0}	$w_{Ta^{3+}}$	$w_{Ta^{1+}}$	$w_{Ta^0_{int}}$	w_{Ta^0}
Samples not triacid treated	[0, 3]	[0, 4]	[0, 5]	[0, 6]	[0.05, 1.5]	[0.05, 1.5]	[0.05, 1.5]	[0.05, 1.5]
Triacid treated sample	[2, 6]	[0, 6]	[0, 7]	[0, 8]	[0.05, 1.5]	[1.05, 2.5]	[1.05, 2.5]	[2.05, 3.5]

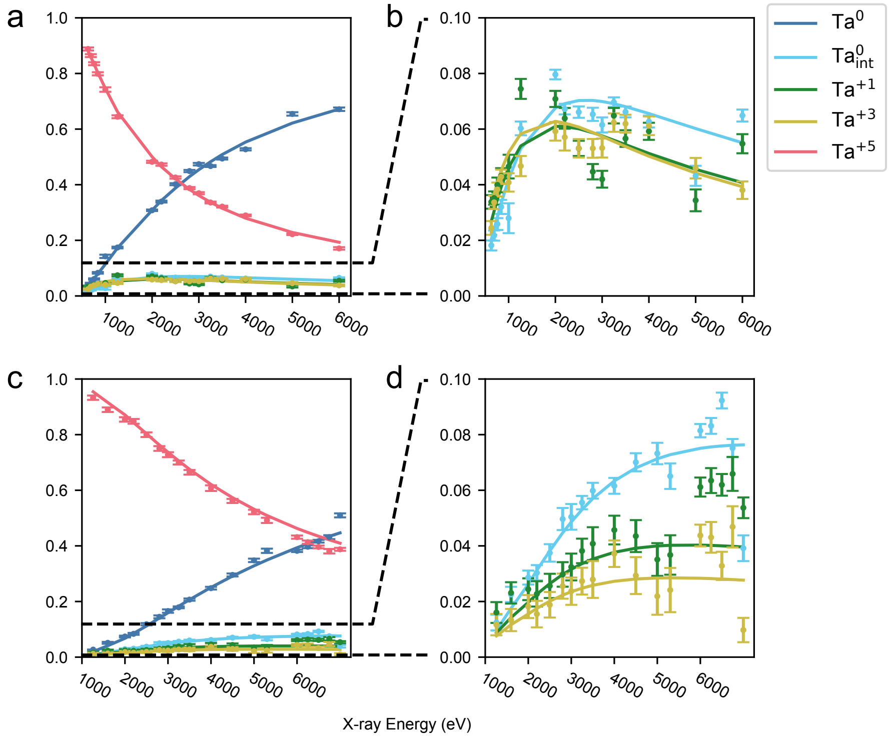

Figure S8: Experimental and simulated photoelectron intensities for the BOE treated (a-b) and triacid treated (c-d) samples. These fits correspond to the depth profiles shown in the main text in Figure 4b and Figure 4c respectively.

S4.5 Uncertainties in effective thickness

Uncertainties for the effective thicknesses of surface species are given in Table 1 in the main text and Table S3. As the effective thickness is not a parameter that is fit in the model, we calculate these uncertainties using a post-fit Monte-Carlo method.

For each sample, we perturb the set of best fit parameters by adding a set of numbers drawn from uncorrelated uniform distributions. We either accept or reject the perturbed parameters based on the corresponding χ^2 value. After we have accepted 100 perturbed parameters, we take the standard deviation of the set of 100 calculated effective thicknesses to report the 1σ uncertainty.

The criteria for accepting a perturbed set of parameters is whether, given our data, the likelihood of the parameters exceeds 0.3. We calculate likelihoods from each parameter set from the χ^2 distribution for our fit; χ^2 values and probabilities are scaled such that the best fit has the maximum probability at 1.

S4.6 Results of fitting algorithm

The fitted photoelectron intensity fractions for the untreated ("Native") tantalum are shown in the main text in Figure 3, and the depth profile is shown in Figure 4a. Depth profiles for the BOE treated and triacid treated tantalum samples are shown in the main text in Figure 4b and Figure 4c, respectively. The fitted photoelectron intensity fractions for these latter two samples are shown in Figure S8.

S5 Peak assignment

As described in the main text, we observe 5 Ta4f doublets. The pair of peaks at 22 eV and 24 eV binding energy has previously been assigned as Ta^0, the pair at 27 eV and 29 eV assigned to Ta^{5+}, and the pair at the shoulder of the Ta^0 peaks assigned as a tantalum species at a material interface with a differing coordination number [6]. The Ta^{5+} in our sample is in the form Ta_2O_5. The other two doublets have binding energies at 23 eV and 25 eV, and 24 eV and 26 eV. We assign these two intermediate doublets as Ta^{1+} and Ta^{3+} based on their similarity to the peak locations reported in [6].

The binding energy position of the Ta^{1+} and Ta^{3+} peaks indicates their charge state, but does not indicate to which chemical compound they belong. A wide survey scan of the untreated ("Native") sample did not indicate any major elements other than tantalum, oxygen, and carbon. We wanted to rule out the presence of nitrides, so we performed a fine scan on the nitrogen KLL Auger line and did not see a peak. The usual line for nitrogen, N1s, overlaps with the $Ta4p_{3/2}$ line.

To rule out that the Ta^{1+} and Ta^{3+} peaks are carbides, we performed an experiment on a separate film using a ThermoFisher K-Alpha X-Ray Photoelectron Spectrometer with an Ar^+ ion gun. We scanned the Ta4f and C1s peaks, then sputtered the film, and then scanned again. The manufacturer provided a calibrated rate curve for sputtering Ta_2O_5, from which we estimate our etch removed approximately 1 nm from the surface of the film. Before sputtering, we observed similar C1s, O1s, and Ta4f spectra to what we observed in the VEXPS dataset at similar X-ray energies. After sputtering, we observe no C1s peak, little change to the O1s peak, a smaller Ta^{5+} peak, non-zero intensity between the Ta^0 and Ta^{5+} peaks (the regions in which the Ta^{1+} and Ta^{3+} peaks are located). These results are shown in Figure S9. We conclude that the Ta^{1+} and Ta^{3+} oxidation states are in the forms of amorphous Ta_2O and amorphous Ta_2O_3.

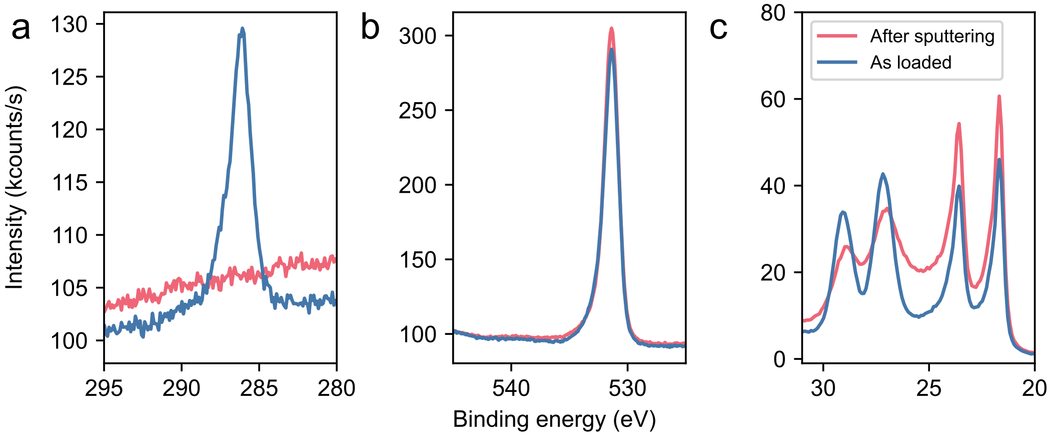

Figure S9: a) C1s spectra for a tantalum film before and after Ar ion milling. The carbon signal is gone after milling. b) O1s spectra for a tantalum film before and after Ar ion milling. Little change is observed. c) Ta4f spectra of tantalum film before and after Ar ion milling. Based on tool calibration, we approximately etched away 1 nm of Ta_2O_5. Some Ta^{5+} signal remains after milling. The photoelectron contribution from tantalum oxidation state(s) with binding energies between the Ta^{5+} and Ta^0 peaks remain. These results indicate that these intermediate binding energy compounds are not carbides.

S6 Effect of piranha clean

All samples underwent piranha cleaning (outlined in Section S2) before VEXPS measurement or further chemical processing, matching the process used in [7], where all devices were piranha

cleaned prior to measurement or surface processing. To characterize the effect this piranha clean has on the tantalum surface, we performed XPS with a ThermoFisher K-Alpha X-Ray Photoelectron Spectrometer on a tantalum film before and immediately after a piranha clean, as well as after a subsequent 20 minute BOE treatment, matching the processing of the "BOE" surface studied in the main text.

In a wide survey scan, we observed a small Na1s peak, but this peak disappears after the piranha clean. The Ta4f spectrum shows a small increase in the intensity of the Ta^{5+} doublet after piranha cleaning, followed by a decrease after the BOE treatment (Figure S10(a)). We also observe an increase in the O1s spectrum intensity after piranha and a decrease after the BOE treatment (Figure S10(b)). The C1s spectrum, by contrast, shows a marked decrease in intensity after piranha, and a further decrease after BOE treatment (Figure S10(c)).

We attribute the Na1s peak seen in the large survey scan to contamination in the lab. Both the Ta4f and O1s spectra indicate a small increase in the oxide thickness after piranha cleaning, and the measurements taken after the 20 minute BOE treatment indicate that the oxide thickness is still larger than when the film was freshly sputtered. The intensity of the C1s peak shows the effectiveness of the piranha clean at removing hydrocarbons from the surface of the sample. The further decrease in C1s intensity after the BOE treatment may be due to the hydrocarbons lifting off as the oxide is etched. We note that the C1s peak intensity in this measurement is generally larger than those measured with VEXPS; as the carbon is entirely adventitious (Section S5), variations in the environment and time between cleaning and measurement could have a significant impact on the signal.

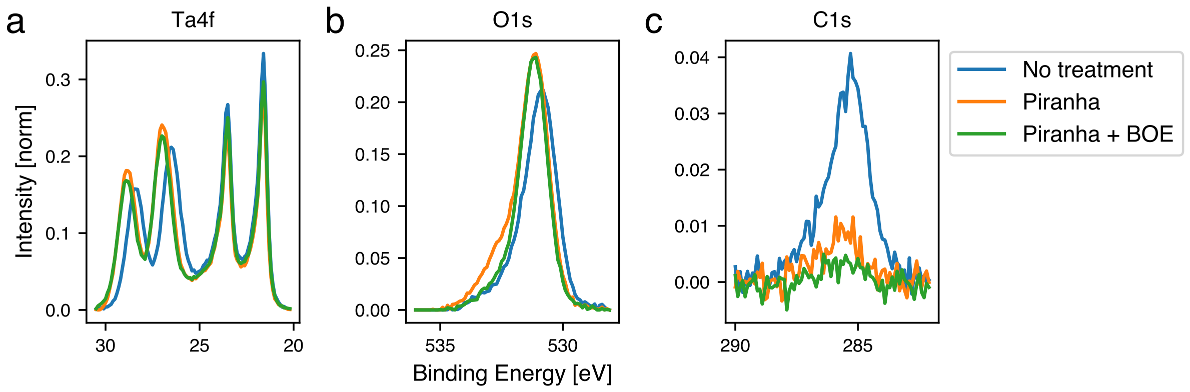

Figure S10: XPS measurements of the Ta4f (a), O1s (b), and C1s (c) spectra, taken on a tantalum sample before piranha cleaning, after piranha cleaning ("native" surface), and after both piranha cleaning and a 20 minute BOE treatment ("BOE" surface). Ta4f and O1s data are Shirley background corrected [2] and the C1s spectra have linear backgrounds subtracted. All spectra on a sample are normalized so the total intensity under the Ta4f spectrum for that sample is unity.

S7 Effect of multiple BOE treatments

In Figure 4 in the main text, we show that the BOE treatment affectw not just the Ta^{+5}, but also the Ta^{+1}, Ta^{+3}, and Ta^{0}_{int} species, which do not appear to be exposed on the surface of the material. One hypothesis that can explain how a BOE surface treatment affects the interface species between the Ta^{0} and Ta^{5+} species is that the BOE treatment strips away all of the Ta_2O_5, interacts with the underlying layers, and then the Ta_2O_5 layer grows back when the sample is exposed to air after the treatment.

To test this hypothesis, we performed VEXPS on two samples from a tantalum film. Both

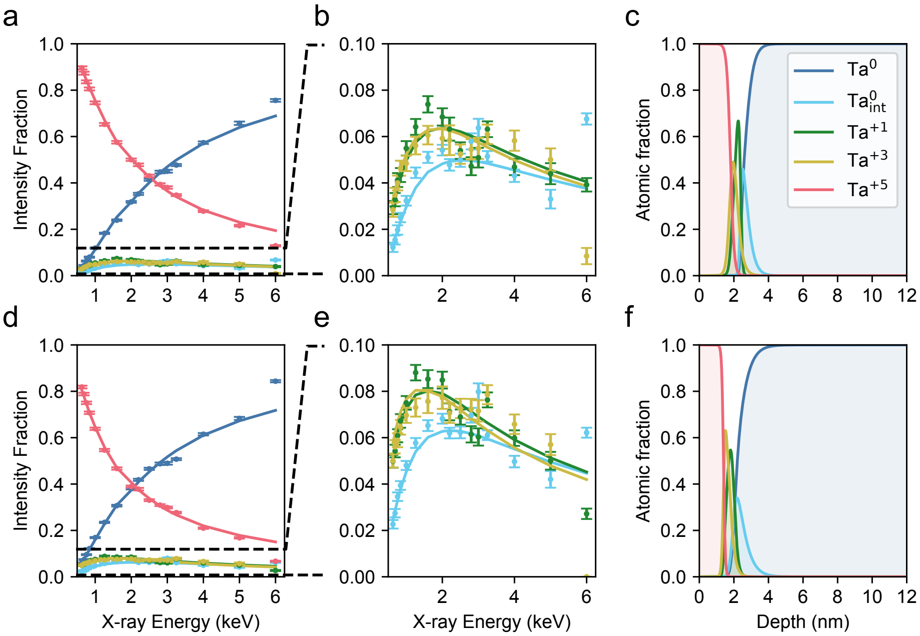

Figure S11: Depth profile fit results for the BOE-1x and BOE-2x samples. a-b) Experimental and simulated relative photoelectron intensities as a function of incident X-ray energy for BOE-1x. c) Fitted interface depth profile for BOE-1x. d-f) as a-c, but for BOE-2x.

samples were treated two weeks before the VEXPS measurement, and one was treated again in BOE immediately before the VEXPS measurements. We denote these two samples as BOE-1x and BOE-2x, respectively. We analyzed the VEXPS data from these samples in the same manner as the native, BOE treated, and triacid treated samples in the main text. The results of this analysis are shown in Figure S11, and the effective thickness of the Ta^{5+}, Ta^{3+}, Ta^{1+}, and Ta^0_{int} species are shown along with those of the native, BOE treated, and triacid treated samples from the main text in Table S3.

The Ta^{5+} thickness of the BOE-1x samples and the BOE treated sample from the main text are similar, both being approximately 0.4 nm smaller than that of the native sample. The Ta^{5+} thickness of the BOE-2x sample is decreased by approximately another 0.4 nm from that of the singly BOE treated samples. As the effect of etching in BOE is roughly additive, we conclude that the BOE treatment is not completely etching away the Ta_2O_5.

Table S3: Effective thickness of different tantalum oxidation states as obtained from depth profile fitting for different tantalum films. All data in nm. Uncertainties are $\pm 1\sigma$ confidence intervals reflecting uncertainty in the fit.

Film	Ta^{5+}	Ta^{3+}	Ta^{1+}	Ta^0_{int}
Native (main text)	2.257 ± 0.023	0.370 ± 0.016	0.370 ± 0.017	0.368 ± 0.019
BOE (main text)	1.853 ± 0.028	0.296 ± 0.022	0.302 ± 0.023	0.400 ± 0.021
Triacid (main text)	4.826 ± 0.036	0.379 ± 0.016	0.545 ± 0.020	1.198 ± 0.027
BOE-1x	1.824 ± 0.017	0.283 ± 0.017	0.285 ± 0.014	0.382 ± 0.020
BOE-2x	1.430 ± 0.014	0.294 ± 0.017	0.314 ± 0.017	0.302 ± 0.019

S8 Pinholes measured by atomic force microscopy

Figure 4 in the main text shows the BOE treatment affecting the Ta^{3+}, Ta^{1+}, and Ta^0_{int} species, even though these species are not at the surface of the samples. Based on the results in Section S7, we do not believe that the BOE treatment is removing the Ta^{5+} layer and affecting the underlying layers.

We performed atomic force microscopy (AFM) on untreated films using a Bruker ICON3 Atomic Force Microscope. We performed AFM on samples from both the film as the three samples (native, BOE treated, and triacid treated) that were discussed in the main text and a film deposited with the same conditions as the film which the BOE-1x and BOE-2x samples. While untreated samples from both films show noticeably different surface morphologies, the measured surface roughnesses over 500 nm squares are significant compared to the 2.257 nm $\pm$ 0.023 nm thick Ta^{5+} layer we found on our native oxide film with VEXPS (Figure S12. We hypothesize that the observed uneven surface morphologies allow the BOE solution access to the buried interface. We note that the observed surface morphologies do not qualitatively change after treatment in BOE.

We must interpret our fitted depth profiles given the observed surface roughness. The XPS spectra were measured with an X-ray beam area of approximately 47 000 μm^2. This area is far larger than the size of features we resolve in either panel of Figure S12. We interpret our fitted depth profiles as an average species fraction through the depth, with the $x = 0$ depth corresponding to the mean height of our samples.

S9 Disclaimer

Certain commercial equipment, instruments, or materials are identified in this paper in order to specify the experimental procedure adequately, and do not represent an endorsement by the National Institute of Standards and Technology.

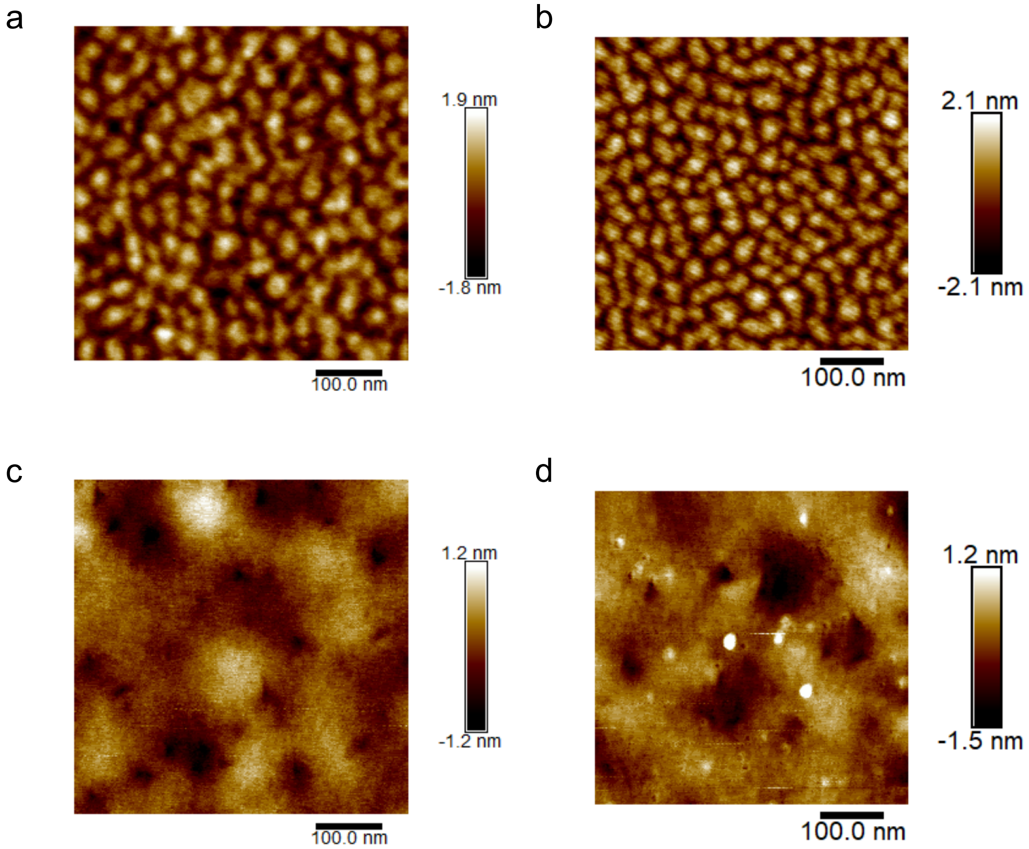

Figure S12: Atomic force microscopy (AFM) image of the height of tantalum samples. a) Untreated sample from the same film as the native, BOE treated, and triacid treated samples described in the main text with extracted root mean square roughness of 0.568 nm. b) as a), but BOE treated with extracted root mean square roughness of 0.648 nm. c) Untreated sample from an identically deposited film as the BOE-1x and BOE-2x samples described in Section S7, with extracted root mean square roughness of 0.324 nm. d) As c), but BOE treated with extracted root mean square roughness of 0.383 nm.

References

[1] D. A. Shirley, *Phys. Rev. B* **1972**, *5*, 12 4709.

[2] M. H. Engelhard, D. R. Baer, A. Herrera-Gomez, P. M. A. Sherwood, *J. Vac. Sci. Technol. A* **2020**, *38*, 6 063203.

[3] P. R. Bevington, R. D. Keith, *Error Analysis*, McGraw-Hill, 3rd edition, **2003**.

[4] URL `https://lmfit.github.io/lmfit-py/intro.html`.

[5] R. Pugliese, G. Paolucci, `https://vuo.elettra.eu/services/elements/WebElements.html`, last accessed on Sept. 2022.

[6] F. J. Himpsel, J. F. Morar, F. R. McFeely, R. A. Pollak, G. Hollinger, *Phys. Rev. B* **1984**, *30* 7236.

[7] K. D. Crowley, R. A. McLellan, A. Dutta, N. Shumiya, A. P. M. Place, X. H. Le, Y. Gang, T. Madhavan, N. Khedkar, Y. C. Feng, E. A. Umbarkar, X. Gui, L. V. H. Rodgers, Y. Jia, M. M. Feldman, S. A. Lyon, M. Liu, R. J. Cava, A. A. Houck, N. P. de Leon, Disentangling Losses in Tantalum Superconducting Circuits, **2023**, (Preprint) arXiv:2301.07848, submitted: Jan **2023**.
